# Kinetic, genomic, and physiological analysis reveals diversity in the ecological adaptation and metabolic potential of *Brachybacterium equifaecis* sp. nov. isolated from horse feces

**DOI:** 10.1128/spectrum.05048-22

**Published:** 2023-09-14

**Authors:** Adeel Farooq, Myunglip Lee, Saem Han, Gi-Yong Jung, So-Jeong Kim, Man-Young Jung

**Affiliations:** 1 Research Institute for Basic Sciences (RIBS), Jeju National University, Jeju, South Korea; 2 Department of Marine Life Science, Jeju National University, Jeju, South Korea; 3 Interdisciplinary Graduate Programme in Advance Convergence Technology and Science, Jeju National University, Jeju, South Korea; 4 Mineral Resources Research Division, Korea Institute of Geoscience and Mineral Resources, Daejeon, South Korea; 5 Department of Biological Sciences and Biotechnology, Chungbuk National University, Cheongju, South Korea; 6 Department of Science Education, Jeju National University, Jeju, South Korea; 7 Jeju Microbiome Center, Jeju National University, Jeju, South Korea; University of Minnesota Twin Cities, St. Paul, Minnesota, USA; University of Pennsylvania, Kennett Square, Pennsylvania, USA

**Keywords:** *Brachybacterium equifaecis* JHP9, cellular kinetics, genome distinctiveness, CRISPR-cas system, fermentation, lactic acid bacteria

## Abstract

Brachybacterium species have been identified in various ecological niches and belong to the family *Dermabacteriaceae* within the phylum *Actinobacteria*. In this study, we isolated a novel *Brachybacterium equifaecis* JHP9 strain from horse feces and compared its kinetic, biochemical, and genomic features with those of other *Brachybacterium* strains. Moreover, comparative genomic analysis using publicly available *Brachybacterium* genomes was performed to determine the properties involved in their ecological adaptation and metabolic potential. Novel species delineation was determined phylogenetically through 16S rRNA gene similarity (up to 97.9%), average nucleotide identity (79.5–82.5%), average amino acid identity (66.7–75.8%), and *in silico* DNA-DNA hybridization (23.7–27.9) using closely related strains. This study also presents the first report of the kinetic properties of *Brachybacterium* species. Most of the *Brachybacterium* strains displayed high oxygen (*K*_m(app)_ =1.6–24.2 µM) and glucose (*K*_m(app)_ =0.73–1.22 µM) affinities, which may manifest niche adaptations. Various carbohydrate metabolisms under aerobic and anaerobic conditions, antibiotic resistance, mobile genetic elements, carbohydrate-active enzymes, lactic acid production, and the clustered regularly interspaced short palindromic repeats-Cas and bacteriophage exclusion systems were observed in the genotypic and/or phenotypic properties of *Brachybacterium* species, suggesting their genome flexibility, defense mechanisms, and adaptability. Our study contributes to the knowledge of the kinetic, physiological, and genomic properties of *Brachybacterium* species, including the novel JHP9 strain, which advocates for their tolerant and thriving nature in various environments, leading to their ecological adaptation.

**IMPORTANCE:**

Basic physiological and genomic properties of most of the *Brachybacterium* isolates have been studied; however, the ability of this bacterium to adapt to diverse environments, which may demonstrate its role in niche differentiation, is to be identified yet. Therefore, here, we explored cellular kinetics, metabolic diversity, and ecological adaptation/defensive properties of the novel *Brachybacterium* strain through physiological and comparative genomic analysis. In addition, we presented the first report examining *Brachybacterium* kinetics, indicating that all strains of *Brachybacterium*, including the novel one, have high oxygen and glucose affinity. Furthermore, the comparative genomic analysis also revealed that the novel bacterium contains versatile genomic properties, which provide the novel bacterium with significant competitive advantages. Thus, in-depth genotypic and phenotypic analysis with kinetic properties at the species level of this genus is beneficial in clarifying its differential characteristics, conferring the ability to inhabit diverse ecological niches.

## INTRODUCTION

The vast amount and diversity of bacteria on Earth, together with ever-increasing human exposure, suggest that we will continuously encounter novel bacterial isolates (1). The gut microbiota plays a vital role in the health, metabolism, and overall well-being of the host. Horses belong to a family of herbivorous mammals that possess a certain hindgut (cecum and colon) microbiota, which provide a substantial proportion of energy for horses through fermentation. Furthermore, equine gut microbiota contributes to essential physiological processes such as digestion, nutrient absorption, and immune system development (2, 3). The equine gut microbiota plays a vital role; however, available data and studies on horse microorganisms are limited. Therefore, isolating and characterizing novel microorganisms from the horse gut offer an opportunity to enhance our understanding of the microbial diversity, unique characteristics, and functional capacities associated with equine gut microbiota. Genomic versatility and the consequent physiological properties of novel strains regarding their adaptation to various ecosystems, including the horse gut, are also required.

*Brachybacterium* is a genus of gram-positive, aerobic, rod-shaped, non-spore-forming bacteria belonging to the family *Dermabacteriaceae* (4). *Dermabacteriaceae* also includes three other genera, namely, *Dermabacter*, *Devriesea*, and *Helcobacillus*. Most members of these genera isolated from diverse clinical samples are considered opportunistic human pathogens, with the exception of the genus *Brachybacterium* (5, 6). To date, *Brachybacterium* includes 24 species with validly published names (https://lpsn.dsmz.de/genus/brachybacterium) and has been isolated from different ecological niches (e.g., soil, plants, water, food products, and animal feces) (7). They are rarely isolated from humans, but a recent case report documented a *Brachybacterium* sp. as the causative pathogen of bloodstream infections in humans (8, 9).

Isolation of *Brachybacterium* strains from a wide range of sources demonstrates that they have adapted to diverse environmental conditions. *Brachybacterium* is a heterotroph that must cope with natural fluctuations, such as the limited availability of nutrients and oxygen, to thrive in various environments similar to other heterotrophic bacteria (10). This ability provides an evolutionary advantage that enables them to outcompete their neighbors (11). Although the basic physiological and genomic properties of most *Brachybacterium* isolates have been studied (7, 12 – 14), the features that contribute to niche differentiation remain unknown. The survival, diversity, and lifestyle strategies of microorganisms largely depend on their encoded ecophysiological repertoires, genomic plasticity, and cellular kinetic properties, including substrate and oxygen affinities (15). The kinetic affinity of a microorganism can be expressed using Michaelis-Menten kinetic equations, analogous to enzyme kinetics, defined by an apparent half-saturation concentration (*K*_m(app)_) and maximal reaction rate (*V*_max_). Based on their cellular kinetic properties*,* microorganisms can be explained in terms of niche differentiation as either growth rate-optimized (*r-*strategists) or growth yield-optimized (*K*-strategists) ecotypes. Thus, in-depth genotypic and phenotypic analyses of the kinetic properties of this genus at the species level will be beneficial for clarifying its differential characteristics that confer the ability to inhabit diverse ecological niches.

Consequently, this study aimed to determine the physiological, kinetic, phylogenetic, and genomic properties of *Brachybacterium* species, particularly emphasizing those of a novel *B. equifaecis* JHP9 strain isolated from horse feces. We investigated the potential adaptability of *Brachybacterium* species to natural habitats by employing comparative genomics of seven *Brachybacterium* genomes and conducting physiological analyses using reference genomes. We focused on elucidating their metabolic potential, particularly their use of various carbohydrates, adaptive strategies, and cellular kinetics. In this study, we sought to enhance our understanding of *Brachybacterium* species and their ecological significance. Specifically, by characterizing the features of strain JHP9, we aimed to provide valuable insights into its adaptative capabilities, potential applications, and the environmental role for its host, the horse.

## RESULTS

### Identification of novel strain JHP9

**Fig 1:**
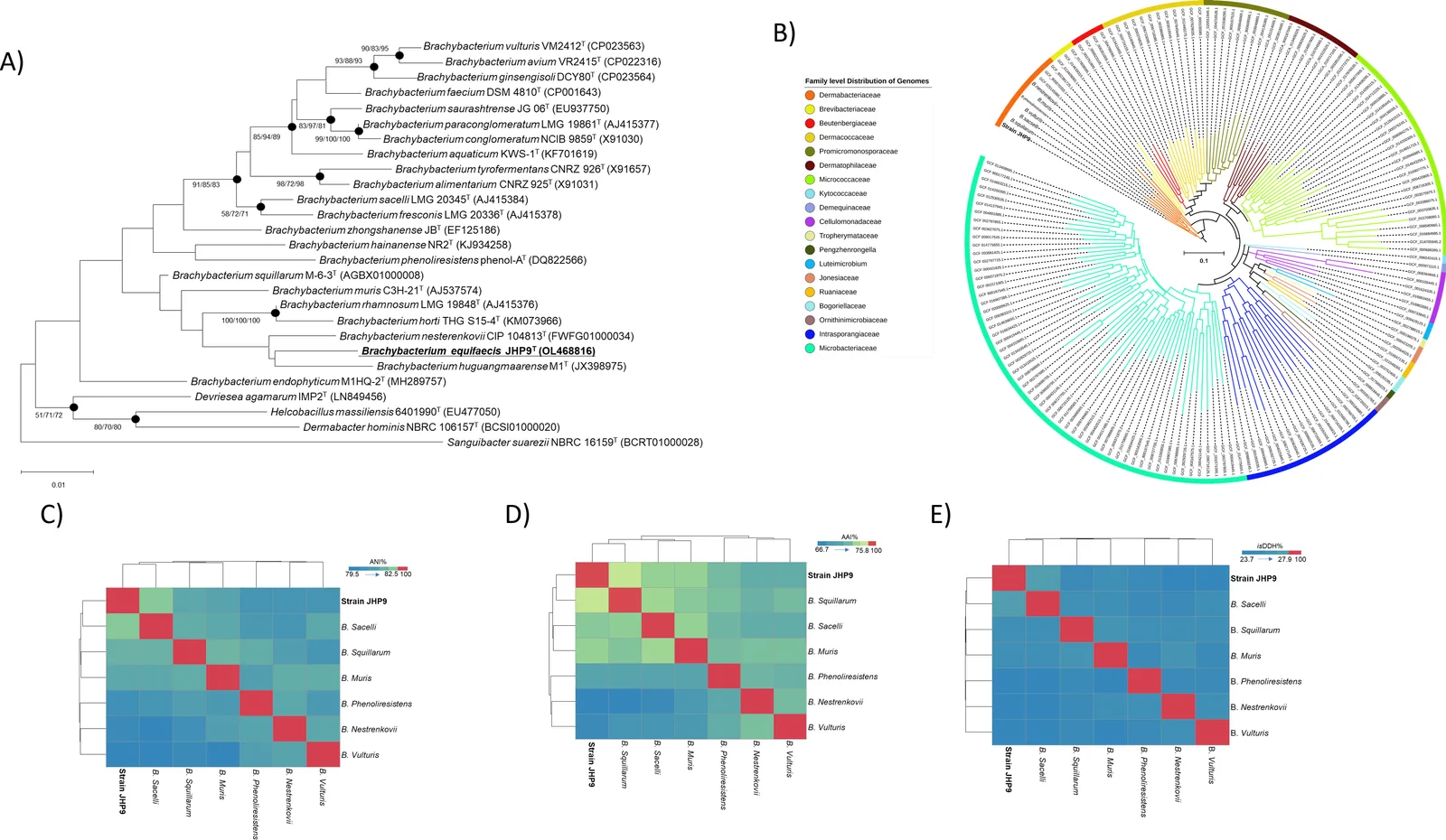
Species delineation and phylogenetic clustering of *B. equifaecis* strain JHP9. (A) Maximum-likelihood tree based on the 16S rRNA gene sequences. Maximum-likelihood, neighbor-joining, and maximum parsimony algorithms with 1,000 replicates were performed for JHP9 and its closely related strains, with the number provided in parenthesis. Filled circles corresponding to nodes were also reconstructed using both neighbor-joining and maximum parsimony algorithms. *Sanguibacter suarezii* NBRC 16,159T (BCRT 01000028) was used as an outgroup. Bar indicates 0.01 substitution per nucleotide position. (B) Phylogenetic tree based on the core genome alignment of the JHP9 genome and its closely associated genomes from the phylum *Actinomycetota*. Representative genomes (163) from 19 different families of the order Micrococcales, along with *Brachybacterium* genomes (7), were downloaded from the NCBI RefSeq database, and alignment was performed using an alignment-free, composition vector-based method through the CVTree4 program. Phylogenetic clusters were visualized using iTOL v.6.5.2. The phylogenetic tree is rooted in strain JHP9, and the branch length depicts the phylogenetic distance. Similarity matrix along with the dendrograms for pairwise genome comparisons of *Brachybacterium* genomes based on (C) average nucleotide identity (ANI), (D) average amino acid identity (AAI), and (**E**) *in silico* DNA-DNA hybridization (*is*DDH).

Phylogenetic analysis was performed based on the 16S rRNA gene sequence to identify the *B. equifaecis* JHP9 strain. Similarities of the closest relatives of this strain were identified as *B. nesterenkovii* CIP104813^T^ (97.9%), *B. rhamnosum* LMG19848^T^ (97.7%), *B. squillarum* THG S15-4^T^ (97.5%), and *B. huguangmaarense* M1^T^ (97.4%) and their phylogenetic locations reconstructed (Fig. 1A). Moreover, whole-genome phylogenetic analysis based on the core genomes of the *Brachybacterium* strains and their closely associated genomes, which was implemented using the composition vector method through the CVTree4 program, exhibited distinctive clustering of the *Brachybacterium* genomes belonging to the family *Dermabacteriaceae* based on phylogenetic distance (Fig. 1B). Among 19 families of the order *Micrococcales*, genome-based clustering revealed a close phylogenetic association between *Dermabacteriaceae* and the *Brevibacteriaceae* and *Beutenbergiaceae* families.

The genome size of strain JHP9 was 3.08 Mbp, with a 71.1% G + C content. It harbored 2,720 protein-coding sequences, 57 RNAs, and an N50 value of 754,379 bp (Table S1). Completeness of the JHP9 genome was 95%, as observed by predicting complete single-copy benchmarking universal single-copy orthologs (BUSCOs) in the genome. These genomic characteristics are nearly in agreement with those of known *Brachybacterium* spp. (Table S1). DNA relatedness has been used as a genotypic parameter to delineate species. Structural analysis of the genome provides insights into its distinctive features. The similarity matrix calculated through comparative genome analysis showed 79.5–82.5% average nucleotide identity (ANI), 66.7–75.8% average protein identity (AAI), and 23.7–27.9 *in silico* DNA-DNA hybridization (*is*DDH) values (Fig. 1C). According to the suggested cutoff values of the ANI (<95–96%) (16, 17), AAI (<95–96%) (18, 19), and *is*DDH (70%) (18) for species delineation, the calculated values based on the comparative genome analysis results indicate that strain JHP9 is distinguished from other previously reported *Brachybacterium* species. The most closely associated strain in terms of the ANI (82.52%) and *is*DDH (27.90%) values was *B. sacelli*, whereas on the basis of the AAI value (75.82%), *B. squillarum* was the closest neighbor of strain JHP9. Genome-based phylogenetic analyses also confirmed the distinctiveness of strain JHP9 and revealed that *Brachybacterium* genomes are closely associated with members of *Brevibacteriaceae*, a family of the phylum *Actinomycetota*, as shown in Fig. 1. Taken together, based on physiological attributes, phylogenetic analysis of the 16S rRNA gene, genome-based phylogenetic analyses, as well as the ANI, AAI, and *is*DDH values, strain JHP9 can be proposed as a representative novel species of the genus *Brachybacterium* with the name *B. equifaecis* sp. nov.

### Morphological, physiological, and biochemical characterization

Cells of the novel JHP9 strain were gram positive, non-motile, and ellipse shaped, having a diameter length of 0.8 × 1.8 µm, as shown via transmission electron microscopy (TEM) (Fig. S1). Structural observations confirmed the rod-shaped morphology of strain JHP9 during its exponential growth phase, whereas it was coccoid during the stationary phase. This behavior was similar to that of *B. nesterenkovii* (20). Colonies were cream colored, smooth, and glistening and grew well on tryptone soya agar (TSA) incubated at 30°C for 7 d. Growth of strain JHP9 occurred at 18–37 °C (optimum, 30°C), pH 6.0–9.0 (optimum, pH 7.0), and 0–10% (wt/vol) NaCl (optimum, 0.5–1%) (Table 1). The strain hydrolyzed starch and DNA but not casein, cellulose, Tween 20, and Tween 80. The hydrolytic activity of *β*-glucosidase was positive but negative for protease, which was the same for all the tested *Brachybacterium* species (Table S2). Positive reactions were detected in the catalase but not in the oxidase tests. Strain JHP9 was negative for indole formation, the dissimilatory reduction of nitrate to nitrite and dinitrogen, and urease activity. The unique enzymatic activity of strain JHP9 against alkaline phosphatase and *β*-glucuronidase was identified (Table S2). All three tested *Brachybacterium* species were positive for the assimilation of d-glucose, *N*-acetyl-d-glucosamine, and gluconate but negative for l-arabinose and caprate (Table S2). The following substrates were used for fermentation: d-ribose, d-galactose, d-glucose, d-mannose, methyl α-d-glucopyranoside, esculin, d-cellobiose, d-lactose, d-trehalose, d-raffinose, and glycogen (Table S2). Among these substrates, d-ribose, d-galactose, d-glucose, d-mannose, and esculin could also be used as universal fermentation sources for other *Brachybacterium* strains, but *N*-acetyl-d-glucosamine was unique only to strain JHP9 (Table S2).

**TABLE 1:** Phenotypic features of the strain JHP9 and its closely associated *Brachybacterium* species^*f*^

Characteristic	1	2 *^a^* ^,^ *^c^*	3^*b*^
Isolated site	Horse feces	Milk product	Lake sediment
Growth in medium	TSB or MRS	TSB + yeast extract^*e*^	TSA + yeast extract^*e*^
Growth in temperature (°C)			
Growth range (optimum)	18–37 (21)	15–42 (21 – 33)	18–40 (21, 25, 26)
Growth NaCl (wt/vol)			
Growth range (optimum)	0–10 (0.5–1)	0–1 (0–0.5)	0–15 (ND^*d*^)
Growth pH			
Growth range (optimum)	6–9 (7)	6–10 (6 – 9)	4–8 (7, 8)
Oxidase	−	+	−
Catalase	+	+	+
Major menaquinone	MK7	MK7	MK7
Peptidoglycan	Meso-DAP, Ala, Glu	Meso-DAP, Ala, Gly, Asp, Glu	Meso-DAP, Ala, Gly, Asp, Glu
Polar lipid profile	DPG, PG, GL	DPG, PG, GL	DPG, PG, GL, PL
DNA G + C content (mol%)	71	70	71
Macromolecule degradation	Starch, DNA	Starch	ND

^*a*^ Data from (20).

^*b*^ Data from (34).

^*c*^ Data from (4).

^*d*^ ND, no data available.

^*e*^ 0.3% yeast extract in media.

^*f*^ 1, *Brachybacterium equifaecis* JHP9T; 2, *Brachybacterium nesterenkovii* JCM11648T; 3, *Brachybacterium huguangmaarense* JCM30544T. meso-DAP, meso-diaminopimelic acid; DPG, diphosphatidylglycerol; PG, phosphatidylglycerol; GL, glycolipid; PL, phospholipid; +, positive; −, negative.

Two-dimensional thin-layer chromatography (TLC) showed that the major polar lipids in strain JHP9 were phosphatidylglycerol (PG), diphosphatidylglycerol (DPG), and glycerolipids (GL) (Fig. S2). This profile was similar to that of other *Brachybacterium* species (20, 34). Its cell wall peptidoglycan contained *meso*-diaminopimelic acid (DAP), alanine, and glutamic acid as the major amino acids, whereas MK-7 (99.48%) and MK-8 (0.52%) were the predominant menaquinones (Table 1), and C_15:0_ anteiso, C_19:0_ cyclo *ω*8*c,* C_17:0_ anteiso, and C_16:0_ iso were the main fatty acid (>5%) components (Table S3). All three tested *Brachybacterium* species shared C_15:0_ anteiso as the most abundant branched-chain fatty acid. Based on chemotaxonomic analysis, strain JHP9 was proposed to belong to the genus *Brachybacterium*. Carbohydrate fermentation was also observed in the strain, although it was weaker than that observed in *B. nesterenkovii* JCM 11648^T^. Based on the susceptibility test results, all three *Brachybacterium* species were susceptible to ampicillin, gentamicin, streptomycin, and tetracycline. However, these strains were resistant to kanamycin. Additionally, strain JHP9 displayed resistance to chloramphenicol, whereas the remaining strains demonstrated susceptibility to it (Table S4).

### Comparative genomics of *Brachybacterium*

**Fig 2:**
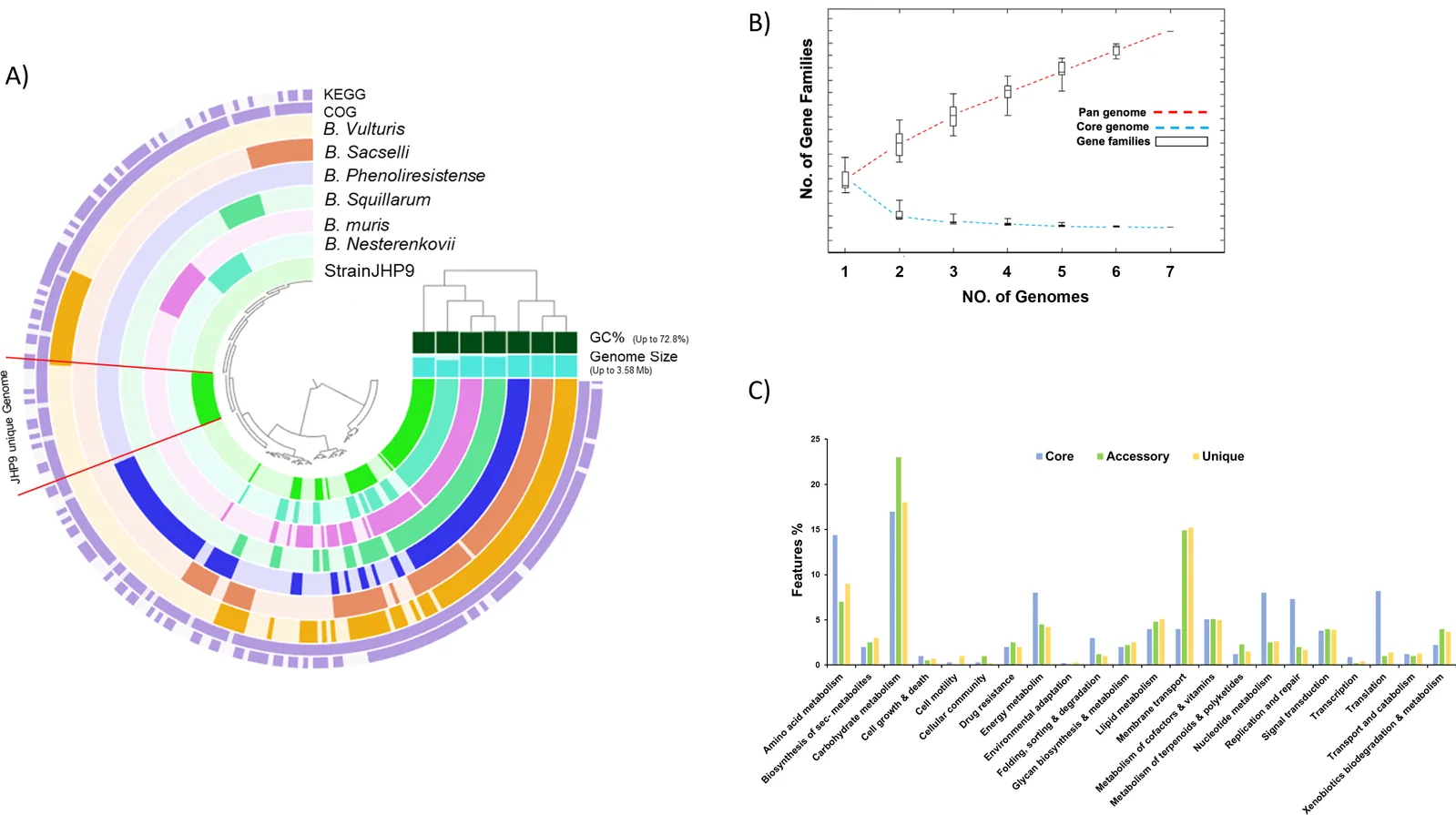
Pangenome analysis of *Brachybacterium equifaecis* strain JHP9. (**A**) Each circle represents a genome, and each radius, a gene family. Core genome families are localized on the right, whereas some of these core families have more than one homologous gene per genome. The shell genome is observed in the middle and at the bottom of the figure, whereas dispensable genome and singletons are represented on the left. Deep ring colors represent the presence of the respective gene clusters, whereas faded ring colors indicate their absence. (**B**) A typical pan/core plot comprised of seven *Brachybacterium* genomes. The core genome decreases when more genomes are added, whereas the pangenome increases upon the addition of genomes. (**C**) Kyoto Encyclopedia of Genes and Genomes (KEGG) distribution of core/accessory/unique genomes in the *Brachybacterium* strains.

A pangenomic analysis of seven *Brachybacterium* genomes, including that of strain JHP9, showed that these organisms share 1,041 core genes. The pangenome comprised 21,264 genes shared among *B. muris* (*n* = 2,736), *B. nesterenkovii* (*n* = 2,455), *B. phenoliresistens* (*n* = 3,509), *B. equifaecis* JHP9 (*n* = 2,660), *B. sacelli* (*n* = 3,902), *B. squillarum* (*n* = 2,679), and *B. vulturis* (*n* = 3,115) (Fig. 2A and B). Functional assignment of the pangenome revealed variations in the distribution of genes within the different metabolic fractions (Fig. 2C). The core genome exhibited a prominent presence in essential processes, such as amino acid metabolism, translation, nucleotide metabolism, replication and repair, energy metabolism, folding, sorting and degradation, cell growth and death, and transcription. In contrast, the accessory genome showed significant representation of functions related to carbohydrate metabolism, cellular community, terpenoid and polyketide metabolism, drug resistance, and xenobiotic biodegradation and metabolism. In contrast, the unique genome demonstrated substantial representation of functions associated with membrane transport, xenobiotic biodegradation and metabolism, lipid metabolism, biosynthesis of secondary metabolites, glycan biosynthesis and metabolism, cell motility and transport, and catabolism (Fig. 2C). These core, accessory, and unique *Brachybacterium* genomes may support their heterotrophic lifestyle, niche differentiation, and adaptation features.

**Fig 3:**
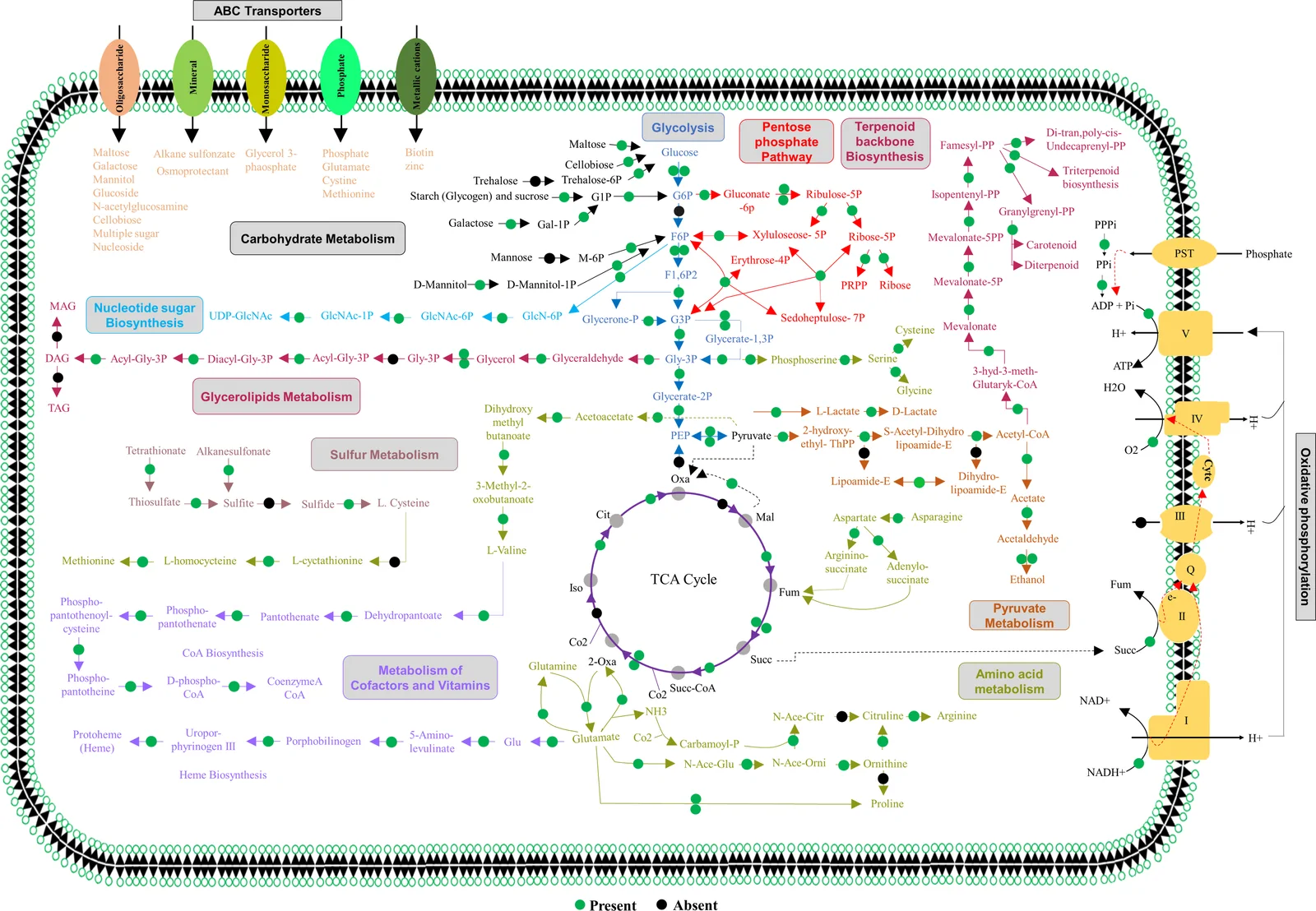
Schematic metabolic potential of the novel *B. equifaecis* strain JHP9. Metabolism pathways were reconstructed based on KEGG annotation. Black dots refer to the absence of respective proteins. The genes that could be detected in strain JHP9 are shown with the green-colored dots. This figure illustrates the genomic potential of strain JHP9 in the uptake of diverse carbohydrates, minerals, and metal ions. It also highlights the involvement of specific proteins in the ABC transportation system for oligosaccharides, monosaccharides, minerals, phosphates, and metallic cations. Additionally, the figure showcases the enzymes associated with the utilization of various carbohydrates, such as galactose, glucose, trehalose, glycogen, lactose, and cellobiose metabolism, as well as pathways such as the pentose phosphate pathway, pyruvate metabolism, amino acid metabolism (arginine, ornithine, proline, and asparagine), cofactors and vitamins (CoA and heme biosynthesis), terpenoid biosynthesis, glycerolipid metabolism, and oxidative phosphorylation.

Thus, the metabolic potential of the novel JHP9 strain was further explored to uncover the complete or nearly complete metabolic pathways encoded by its genome. In-depth analysis revealed that its ABC transportation system consisted of various components, including oligosaccharides (nineeach), phosphates (four), minerals (two), metallic cations (two), and monosaccharides (one) (Fig. 3). Furthermore, metabolic pathways for carbohydrates, such as maltose, cellobiose, starch, galactose, d-mannitol, trehalose, and mannose, were identified within the genome. Additionally, it exhibited the presence of the pentose phosphate pathway, terpenoid backbone biosynthesis, pyruvate metabolism, amino acid metabolism, GL metabolism, nucleotide sugar biosynthesis, and the metabolism of cofactors and vitamins. Genomic analysis of this bacterial strain revealed the presence of pathways related to oxidative phosphorylation, demonstrating its capacity for efficient energy production through aerobic respiration (Fig. 3). Notably, similar metabolic potential patterns were identified in other *Brachybacterium* genomes, except for variations in the presence of ABC transporters (Fig. S6).

Detailed genomic analyses revealed genes coding for antibiotic resistance, the clustered regularly interspaced short palindromic repeats (CRISPR) system, mobile genetic elements (MGEs), carbohydrate-active enzymes (CAZymes), and stress responses (Table 2; Fig. S4; Supplementary File 2). The JHP9 genome was found to carry three antibiotic resistance genes (ARGs) that confer resistance to two distinct groups of antibiotics. These ARGs were *gyrA* (locus number; Bequi_11645) and *gyrB* (Bequi_11640), encoding resistance against quinolones, while *blaIII* (Bequi_03615) gene determining resistance against beta-lactam antibiotics. In contrast, none of the other *Brachybacterium* genomes carried any of the known resistance determinants. Moreover, no virulence factors and prophages were detected in the *Brachybacterium* genomes. The Type I-E CRISPR-Cas system was observed in the JHP9, *B. nesterenkovii*, *B. squillarum*, and *B. vulturis* genomes. The CRISPR-Cas locus comprises three arrays and eight associated proteins, including *cas2e*, *cas1e*, *cas6e*, *cas5e*, *cas7e*, *casB, casA*, and *cas3* (Bequi_14035-14070), encompassing the type I-E CRISPR system. The *B. nesterenkovii*, *B. squillarum*, and *B. vulturis* strains also expressed these eight associated proteins; *B. nesterenkovii* had two CRISPR arrays, and *B. squillarum* and *B. vulturis* each had one array, as described in Table 2; Supplementary File 2. Moreover, the bacteriophage exclusion (BREX) system (Bequi_11770-11790) was identified in the JHP9 genome. Several MGEs were found in *Brachybacterium* genomes, including IS30, ISL3, IS256, and IS110, which were distributed over seven, six, six, and six genomes, respectively (Table 2; Supplementary File 2).

**TABLE 2:** Comparative genomic analyses of CAZymes, stress-related genes, CRISPR systems, MGEs, and ARGs encoded by the *Brachybacterium* genomes (*n* = 07)Supplementary file 2^*b*^

Genomes	CAZymes	Stress-related genes	CRISPR system		MGEs (family)	ARGs
			CRISPR arrays	Associated proteins		
Strain JHP9	GT (27) GH (11) CE (4)	SOD (1) GPx (2) Trx (2) CAT (1) Csp (2) BetT (1)	3	8	IS3 (07) IS30 (06) IS481 (06)	Quinolones (2) Beta-lactam (1)
*B. muris*	GT (30) GH (14) CE (1)	SOD (1) Trx (2) CAT (1) Csp (3) BetT (1) EctC (1)	ND^*a*^	ND	IS21 (135) IS3 (18) IS1380 (14)	ND
*B. nesterenkovii*	GT (30) GH (15) CE (2)	SOD (1) GPx (1) Trx (2) CAT (1) Csp (2) BetT (1) EctC (1)	2	8	IS110 (9) ISL3 (8) IS256 (5)	ND
*B. phenoliresistens*	GT (29) GH (30) CE (1)	SOD (2) GPx (2) Trx (1) CAT (2) Csp (3) BetT (1) EctC (1)	ND	ND	IS110 (03) IS30 (1)	ND
*B. sacelli*	GT (25) GH (34) CE (0)	SOD (3) GPx (2) Trx (1) CAT (2) Csp (3) BetT (1) EctC (2)	ND	ND	IS3 (20) IS481 (11) IS256 (06)	ND
*B. squillarum*	GT (32) GH (13) CE (2)	SOD (1) GPx (2) Trx (1) CAT (1) Csp (3)	1	8	IS30 (7) IS481 (6) IS110 (5)	ND
*B. vulturis*	GT (22) GH (13) CE (1)	SOD (2) GPx (1) Trx (1) CAT (1) Csp (3) BetT (2) EctC (2)	1	8	IS110 (6) IS1380 (5) IS30 (2)	ND

^*a*^ ND, not detected.

^*b*^ The type and number of attributes are given here, while a detailed description is given in Supplementary file 2. GT, glycosyl transferases; GH, glycoside hydrolases; CE, carbohydrate esterases; SOD, superoxidase dismutase; GPx, glutathione peroxidase; Trx, thioredoxin; CAT, catalase; CspA, cold shock protein; BetT, choline transporter; EctC, ectoine synthase; T3PKS, 5-acetyl-5,10-dihydrophenazine-1-carboxylic acid.

Based on CAZyme analysis, *Brachybacterium* genomes (*n* = 7) contained a number of carbohydrate-active genes coding for glycosyl transferases (GTs) (21 – 32), glycoside hydrolases (GHs) (11 – 20, 34), and carbohydrate esterases (CEs) (0–4), indicating their ability to utilize a variety of carbohydrates (Table 2; Supplementary File 2). Similar to most of the *Brachybacterium* strains, strain JHP9 has canonical defense systems that aerobic organisms need to survive against oxidative stress, including superoxide dismutase, catalase, thioredoxin, and glutathione peroxidase (Supplementary File 2). Moreover, most *Brachybacterium* genomes contained genes related to oxidative and osmotic stress, such as cold shock proteins and choline (BetT) biosynthesis (Table 2 and Supplementary File 2).

Furthermore, genome annotation revealed that JHP9 and other *Brachybacterium* genomes encode genes for two types of terminal oxidases, including cytochrome *d* ubiquinol subunits I and II (CydAB), which predominate under low-aeration growth conditions with high oxygen affinity. Cytochrome *c* oxidase subunits I, II, and IV (COX1, 2, and 4) are heme-copper oxidases (HCO) that prefer high-aeration conditions with low oxygen affinity (35). The *cydA* and *cydB* genes in the cytochrome *d* ubiquinol cluster encode two polypeptide subunits of the cytochrome *d* terminal oxidase complex, whereas *cydC* and *cydD* are involved in the assembly of cytochrome *bd*-I, a terminal oxidase of the respiratory chain required for growth under low oxygen conditions. A cluster of these four genes, which constitutes cytochrome *d* ubiquinol, was encoded by all *Brachybacterium* genomes (Fig. S5). Moreover, a COX cluster comprising the *cox1*, *cox2*, and *cox4* genes was present in all *Brachybacterium* genomes, as shown in Fig. S5.

### Lactic acid production by *Brachybacterium*

**Fig 4:**
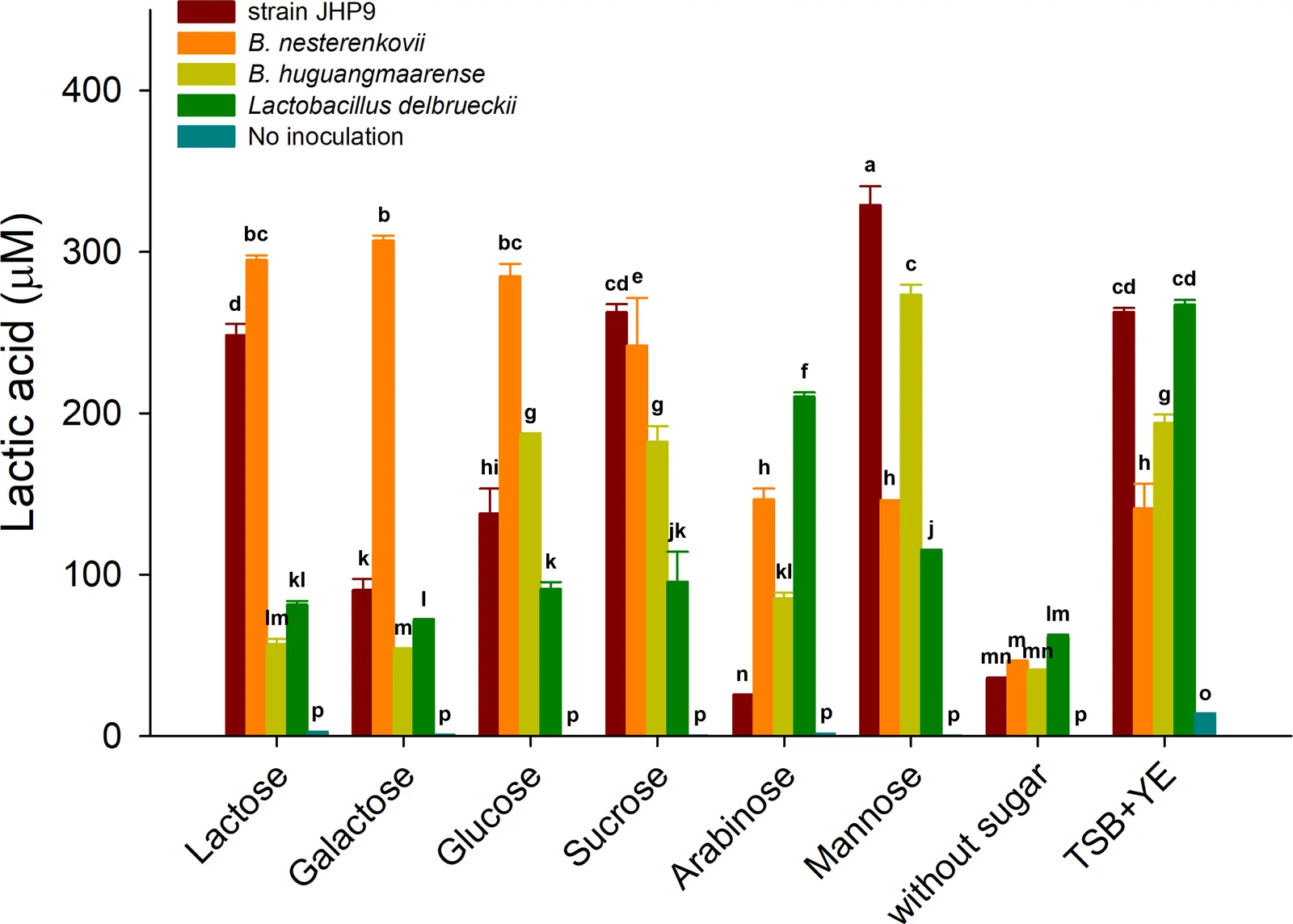
Lactic acid production of three *Brachybacterium* strains. A lactic acid bacterial strain, *Lactobacillus delbrueckii* subsp. *bulgaricus* KCTC 3769^T^, was also tested for comparison. Artificial freshwater medium without sugar and no inoculum served as a negative control. Error bars represent the standard deviation for *n* ≥ 3 biological replicates. Significant differences between treatments in each strain are indicated by different letters (one-way analysis of variance, Tukey’s test, *P* < 0.05).

Quantitative analysis of lactic acid production during fermentation by strain JHP9 and its closely associated strains was performed using six different sugar compounds. Our results revealed a significant difference in lactic acid production between the *Brachybacterium* strains, and strain JHP9 produced a higher concentration of lactic acid from mannose and sucrose than that of other *Brachybacterium* strains (Fig. 4). In contrast, *B. nesterenkovii* JCM11648^T^ produced the highest lactic acid concentration from galactose, lactose, and glucose. The *B. huguangmaarense* JCM 30544^T^ strain only produced a significant concentration of lactic acid from mannose, glucose, and sucrose. The results of lactic acid production were in accordance with those of the fermentation activity (Fig. 4; Table S2), except for some sugars, such as galactose, which was positive and did not produce lactic acid. Notably, under our experimental conditions, the lactic acid-producing bacterium (LAB), *Lactobacillus delbrueckii* subsp. *bulgaricus* KCTC 3769^T^, used as a positive control strain, did not produce higher levels of lactic acid, except when arabinose was used as a substrate (~210 µM).

### Substrate and oxygen affinity in *Brachybacterium*

**Fig 5:**
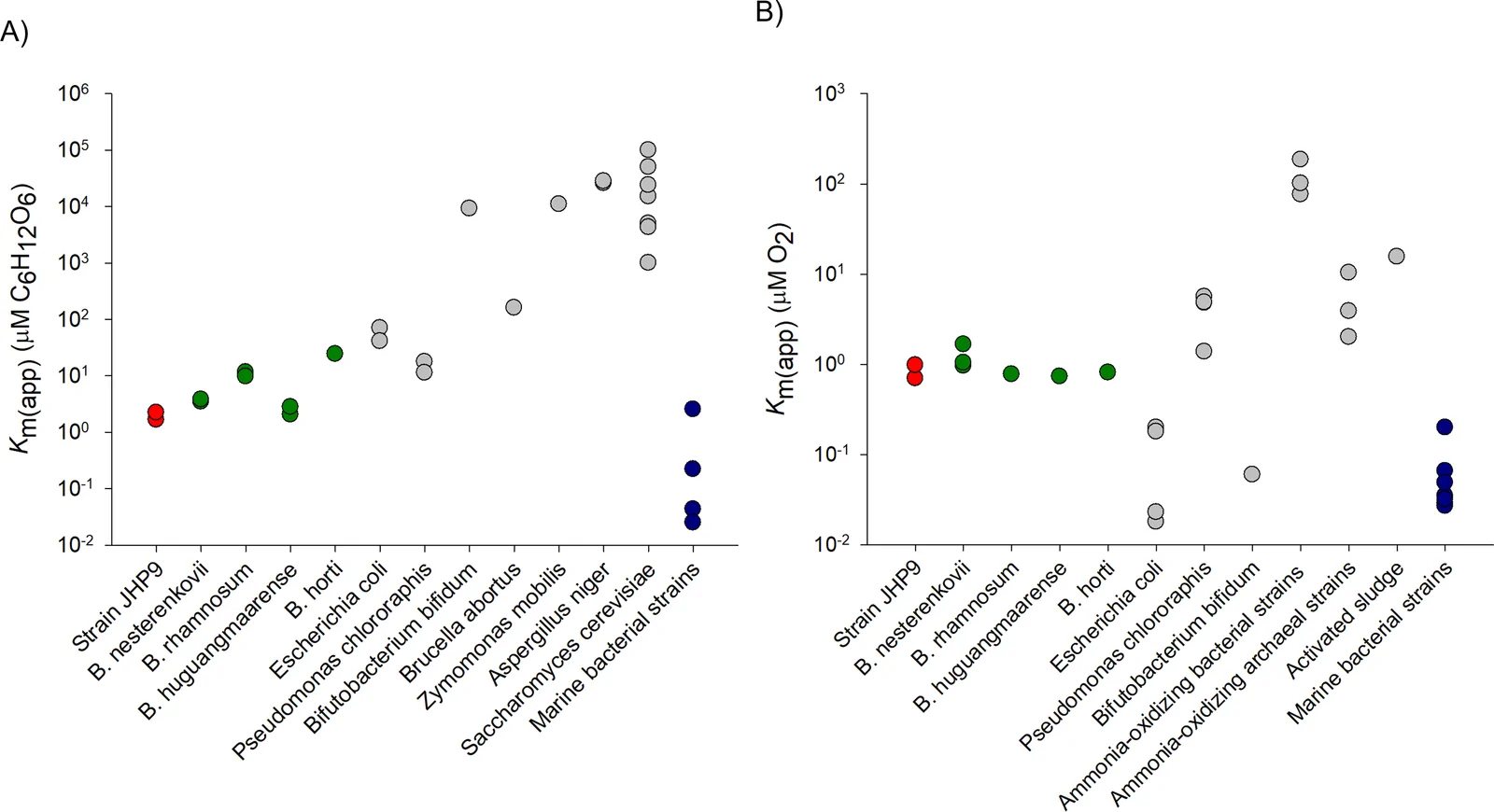
Comparison of the whole-cell apparent half-saturation constants (*K*_m(app)_) for glucose and oxygen between *Brachybacterium* strains and other microbes. (A) Glucose and (B) oxygen uptake affinities are shown with distinctive colors. The *K*_m(app)_ values measured in this study are highlighted in red for strain JHP9 and green for other tested *Brachybacterium* strains. The other *K*_m(app)_ values were retrieved from previous studies (22 – 24, 36 – 41). The individual Michaelis-Menten plots for each strain determined in this study are presented in Fig. S6.

Cellular respiration kinetics for glucose and oxygen affinity were determined using strain JHP9 and other closely associated *Brachybacterium* species by measuring glucose- and oxygen-dependent oxygen consumption in microrespirometry (MR) experiments (Fig. S6). The stoichiometry of glucose (C_6_H_12_O_6_) and O_2_ consumption was always near 1:6 (mean = 1:6.11; SD = 0.12; *n* = 15), which was expected for heterotrophs that could use glucose as a substrate. The apparent *K*_m(app)_ for glucose of strain JHP9 was determined to be 1.9 ± 0.3 µM, which was the highest affinity calculated among all tested *Brachybacterium* strains. The other tested *Brachybacterium* strains, excluding *B. horti* (*K*_m(app)_ =24.26 µM), had a similar affinity range for glucose (*K*_m(app)_ =2.04–10.6 µM). However, the cellular kinetic affinity for oxygen of all tested *Brachybacterium* strains was in a similar range (*K*_m(app)_ =0.73–1.22 µM) (Fig. 5). The oxygen uptake affinity was much higher than that of heterotrophic bacteria (*Escherichia coli* and *Pseudomonas chlororaphis*) (22, 23, 36, 37) and autotrophic ammonia oxidizers (ammonia-oxidizing bacteria and archaea) (24) but lower than that of the LAB strain, *Bifidobacterium bifidum* (25). The affinity for glucose and oxygen in *Brachybacterium* strains was lower than that of the marine bacterial consortium in oligotrophic habitats.

## DISCUSSION

In this study, we described the phenotypic and genotypic characteristics of a novel *B. equifaecis* JHP9 strain, along with those of closely related *Brachybacterium* strains, using physiological and genomic analyses. Interestingly, *Brachybacterium* species possess properties that facilitate their versatile lifestyles and ecological adaptations. These characteristics encompass their ability to survive under various temperature, pH, and salt concentration conditions, their ability to utilize a variety of carbohydrates, and their high affinity for oxygen and glucose. Collectively, these traits facilitate successful adaptation and survival in specific environmental settings. Furthermore, analysis of the novel JHP9 strain revealed unique factors contributing to its defense mechanism, including antibiotic resistance, relatively high affinity for oxygen and glucose, and efficient utilization of sugars. These traits reflect the adaptation of strain JHP9 to the niche environment of the horse intestine.

According to the competitive exclusion principle of ecology, two species competing for the same limiting resource cannot coexist under stable environmental conditions (26). However, the co-occurrence of microbes competing for the same substrate has been verified in various natural and engineered environments. This observation challenges the traditional notion of competitive exclusion and prompts inquiries into the physiological traits that enable niche adaptation within microbial communities. For heterotrophic bacteria, such as *Brachybacterium*, efficient utilization of substrates (carbohydrates) requires the metabolic potential to obtain energy from available resources (21).

Carbohydrate utilization involves the hydrolysis of complex polysaccharides, which requires multiple CAZymes owing to the structural diversity of carbohydrates (27). *Brachybacterium* genomes contain genes encoding GTs, CEs, and GHs that hydrolyze cellular carbohydrates (28). The prevalence of these enzymes enables *Brachybacterium* strains, including JHP9, to efficiently utilize diverse carbohydrates as a competitive advantage against bacterial species using the same substrate (29). Although we did not experimentally validate specific functions of the predicted CAZymes, their ability to produce lactic acid from five different sugars (Fig. 4), assimilate five different carbon sources, and ferment 11 distinctive carbohydrates (Table S2) implies their potential involvement in carbohydrate utilization. Nevertheless, it is crucial to acknowledge that the roles of CAZymes in sugar fermentation can differ depending on the bacterium and its metabolic capabilities.

Functional annotation of the genomes revealed the presence of proteins involved in carbohydrate metabolism, including glycolysis and the tricarboxylic acid cycle, which are crucial components of the central carbon dissimilation pathway. *Brachybacterium* genomes encoded nearly all the genes required for various metabolic pathways, including carbohydrate metabolism, membrane transport (ABC transport system), and amino acid metabolism (Fig. 3; Fig. S3). Specifically, the unique *Brachybacterium* genome contained ABC transporter systems that are responsible for transporting oligosaccharides, monosaccharides, minerals, phosphates, and metallic cations (as shown in Fig. 3). This allows *Brachybacterium* spp. to cope with the chemical constraints of their natural habitats, leading to niche adaptations. Additionally, the accessory genome is involved in carbohydrate metabolism (Fig. 2C), suggesting its potential contribution to niche differentiation. Furthermore, the core *Brachybacterium* genome was involved in amino acid metabolism, suggesting that it supports a heterotrophic lifestyle. The utilization of external sources of organic compounds, including amino acids, is vital for their growth and energy production.

Lactic acid production via microbial fermentation offers advantages over chemical synthesis, including simplicity of operation, low substrate costs, moderate pressure and temperature conditions, reduced risk of contamination, low energy consumption, and improved environmental performance (30). Various microbial species, including bacteria, fungi, yeast, cyanobacteria, and algae, produce lactic acid using carbohydrates as the only or primary carbon source (31). LAB strains are predominantly anaerobic and utilize pyruvic acid, an intermediate in the Embden-Meyerhof pathway, as a substrate for lactate production. The conversion of pyruvic acid to lactate is facilitated by the activity of l- or d-lactate dehydrogenase (LDH) enzymes. Notably, *Brachybacterium* genomes also encode enzymes involved in lactate fermentation, which is consistent with the phenotypically expressed fermentation abilities of these strains, as shown in Fig. 4. The genome of strain JHP9 encodes l-LDH (*ldh*; Bequi_03000), d-LDH (*ldhd*; Bequi_04310), and lactate racemase (*larA-C*; Bequi_13030) on distinct contigs, mirroring their presence in other tested *Brachybacterium* strains. This genetic evidence suggests that these enzymes may play a crucial role in producing l- and d-lactic acid through the fermentation processes employed by *Brachybacterium* strains. Under our experimental conditions, the *Brachybacterium* strains produced higher levels of lactic acid than that of the *Lactobacillus* LAB strain. Under various growth conditions, *Lactobacillus* strains may behave as heterofermenters and produce not only lactic acid but also acetate, formate, ethanol, diacetyl, acetoin, and CO_2_ (32). Heterofermentative LAB can use the phosphogluconate and phosphoketolase pathways to metabolize hexose and pentose sugars, respectively. Previously, it was observed that lactic acid production by LAB through glucose consumption was lowest in MRS (De Man–Rogosa–Sharpe) media; instead, high acetate generation was observed (33), which might be due to fluctuating sugar and protein contents of the media and the incubation time. Similarly, the product of d-galactose fermentation in *B. huguangmaarense* was not lactic acid (Fig. 4; Table S2).

Although all the tested *Brachybacterium* strains demonstrated kanamycin resistance, strain JHP9 exhibited phenotypic resistance to chloramphenicol and kanamycin. Interestingly, our analysis revealed quinolone- and ampicillin-resistance determinants in the JHP9 genome, indicating its potential resistance to these antibiotics. However, we could not identify any known kanamycin or chloramphenicol resistance genes in the JHP9 genome. The observed phenotypic resistance may be attributed to mutated genes that are not homologous to the known resistance determinants for kanamycin and chloramphenicol. Alternatively, this could be due to the expression of cryptic genes that were not evaluated in this study. Conversely, the JHP9 genome carried beta-lactam-resistance genes despite exhibiting susceptibility to the corresponding antibiotic when tested phenotypically. The lack of phenotypic resistance associated with the beta-lactam-resistance gene, *blaIII*, may be attributed to several factors. These factors include mutations or genetic variations within the gene or its regulatory elements, absence of specific inducers or repressors, and genetic regulatory mechanisms (42 – 44). Although strain JHP9 harbors quinolone-resistance genes, its phenotypic resistance to antibiotics belonging to this class was not evaluated in this study (Table S4). *Brachybacterium* strains were reportedly resistant to ampicillin, cefazolin, vancomycin, erythromycin, rifampicin, gentamicin, clindamycin, and tetracycline (34, 45). However, this is the first report of chloramphenicol resistance and the identification of quinolone resistance determinants in any *Brachybacterium* species. Resistance to antibiotics provides an advantage for the propagation of strain JHP9, where it can outcompete susceptible isolates in a stressed environment, such as under an antibiotic selective pressure. The possible acquisition of chloramphenicol and quinolone resistance from the host (horse gut) indicates the excessive use of antibiotics to treat infections and for prophylactic purposes in livestock farming (46). Strain JHP9 may spread the antibiotic resistance trait by transferring the encoded ARGs to other closely associated human pathogenic bacteria via horizontal gene transfer.

*Brachybacterium* genomes also contained defense- and stress-related genes, as well as CRISPR-Cas systems, specifically the type I-E CRISPR system, with the JHP9 genome additionally possessing the BREX system. These systems enhance defense and immunity against foreign DNA and lytic and temperate phages (47, 48). Moreover, their genomes contained oxidative and osmotic stress-related genes (Table 2), which could enhance their ability to propagate in various ecosystems. Additionally, the presence of MGEs, including IS3, IS30, and IS481, in the JHP9 genome (Table 2), indicates its genetic variability, which consequently leads to adaptive evolution. Transposable elements are agents of genetic variability in bacteria because they are a source of adaptive evolution through genome diversification (49). In addition, MGEs are thought to enhance the defense mechanisms of bacteria (50). MGEs are known to transpose associated genes within the genome, which not only produces genome diversity, but also enhances the phenotypic expression of colocalized genes (51).

This study is the first to report the glucose and oxygen affinities of any *Brachybacterium* species, including those within the *Dermabacteriaceae* family. All tested strains, including strain JHP9, had a relatively high affinity (with a low *K*_m(app)_ value) for glucose and are comparable with those of *E. coli* (38) and *P. chlororaphis* (23) (see Fig. 5). These traits confer essential competitive advantages to heterotrophic bacteria (39, 40) and likely provide similar benefits to *Brachybacterium* species under oligotrophic conditions, such as in marine bacterial populations with 1–2 orders of magnitude higher glucose affinity (40). Therefore, whether *Brachybacterium* strains can persist at extremely low *in situ* substrate concentrations remains to be tested. For example, highly different substrate affinities have been observed for *E. coli* when grown under oligotrophic versus copiotrophic conditions (41). Therefore, a high glucose affinity might endow various fitness strategies to JHP9 and other *Brachybacterium* strains.

The final step of aerobic respiration involves a terminal oxidase, a membrane-associated protein that reduces O_2_ to H_2_O. Genomic analysis of strain JHP9 revealed the presence of genes encoding terminal oxidases, including two different terminal oxidases (cytochrome *bd* oxidase and HCO), as shown in Fig. S5, which are considered high- and low-oxygen affinity oxidases (36). Cytochrome *bd* oxidases play crucial physiological roles in enabling bacterial survival and reproduction under adverse environmental conditions. Thus, the prevalence of these terminal oxidases in the JHP9 genome not only provides it with a high oxygen affinity, which enhances its adaptability to various niches with diverse living conditions (52) but also enables its survival under toxic conditions. As described above, high- and low-affinity terminal oxidases are classified based on their different O_2_ affinities (36, 53). Strain JHP9 has both high- and low-affinity terminal oxidase genes, similar to *E. coli* and marine bacterial strains, and the high-affinity terminal oxidases may enable these bacteria to maintain high levels of respiratory activity even when the O_2_ concentration decreases. In addition, the oxygen affinity of washed cell suspensions of an LAB strain, *Bifidobacterium*, was high (*K*_m(app)_ =0.06 µM) (25) and likely resulted from the function of a high-affinity cytochrome *d* oxidase (54). Even under high O_2_ conditions, *E. coli* expressed high-affinity terminal oxidases and were incubated under fully aerobic conditions; thus, they may exhibit relatively low *K*_m(app)_ values similar to those of *Brachybacterium* strains. It was suggested that the *K*_m(app)_ value changes were correlated with the availability of an electron donor (55) and those for glucose oxidation affected by the oxygen concentration in *P. chlororaphis* culture; therefore, the oxygen uptake kinetics could be related to substrate affinity (23). It has also been shown that the different cell sizes (with different surface area-to-volume ratios) of various ammonia-oxidizing autotrophs are correlated with their kinetic affinity (15) and show different *K*_m(app)_ values for different cell sizes of a single marine bacterial strain. Therefore, the microbial aerobic respiration rate is affected by various factors, including cell size, physiological growth conditions, nutrient availability, and O_2_ concentration, which eventually control oxygen and substrate affinity. Our results imply that *Brachybacterium,* including strain JHP9, has a high affinity for glucose and oxygen. The *K-strategist* microbes grow slowly but can compete at low substrate concentrations because of their high substrate affinity (39). Therefore, members of the family *Dermabacteriaceae*, including strain JHP9, tend to utilize glucose and oxygen to survive in temperate ecosystems; hence, such a high substrate affinity is advantageous to allow them to adapt and colonize the host while competing with specialists in the same niche (56). Furthermore, the high GC content observed in *Brachybacterium* genomes (Table 1) contributes to the stability of their DNA (44). This characteristic implies that they have the capacity to flourish within specific temperature ranges and adapt to their ecological niches.

The metabolic potential, defense strategies, and physiological characteristics of *Brachybacterium* spp. contribute to their niche adaptation to specific environments, including the unique adaptation of strain JHP9 to horse intestines. High resource diversification can lead to gradual ecological specialization, in which bacterial populations progress toward an optimal phenotype. However, resource or host generalism, which confers ecological advantages, has also been widely observed in nature and confers an ecological advantage (57). The adaptability of *Brachybacterium* strains may be attributed to the development, industrialization, and globalization of livestock farming that has created open niches in which bacteria have expanded their hosts. Livestock husbandry and habitation facilitate close contact between different species, thereby providing opportunities for bacterial transmission from one host to another (58). This transmission drives the acquisition of a broad range of traits that are necessary for environmental adaptation. Strain JHP9 exhibits features such as carbohydrate utilization, defense mechanisms, and affinity for oxygen and glucose, which contribute to its successful adaptation and survival in the horse gut environment.

In this study, we comparatively characterized various *Brachybacterium* species, with a particular focus on *B. equifaecis* JHP9, to assess their ecological adaptation and metabolic potential. However, the physiological properties deduced from *in vitro* and genomic analyses and niche differentiation under actual environmental conditions can be uncoupled. Furthermore, investigations of the genomic and physiological properties of species across a range of ecological factor gradients are essential for understanding the adaptations between species in various environmental systems. Hence, further investigations and ecological experiments, such as *in situ* or microcosm studies with different environmental factor treatments, are necessary to understand the ecological adaptations of bacterial clusters.

### Conclusion

This study indicates that strain JHP9 represents a novel species of the genus *Brachybacterium* based on 16S rRNA analysis, genome-based phylogenetic analyses, and similarity matrix data using the ANI, AAI, and *is*DDH values. Physiological studies uncovered the diverse growth conditions and carbohydrate utilization of the novel strain, along with its chemotaxonomic phenotypes. The phylogenetic relationships among members of the genus *Brachybacterium* were determined to characterize the diversity of this genus. The versatile metabolic potential of the strain JHP9 to utilize various carbohydrates and lactic acid production enables its propagation and potential utilization in the food industry. In addition, this is the first study to report the high oxygen and glucose affinity of strain JHP9, its resistance to quinolone and phenicol antibiotics, and the prevalence of CRISPR systems in its genome, suggesting its versatility, adaptability, and opportunism. Taken together, future in-depth analyses of *Brachybacterium* spp. to uncover their interactions in various environments, genome plasticity, and impact on neighboring cells are required to completely understand their beneficial or harmful dynamics in the natural environment. Further metagenomics-based studies should be conducted to estimate their abundance across various ecosystems to elaborate on their adaptation versatility.

#### Description of *B. equifaecis* JHP9 sp. nov. (L.n. *Equus*, horse; L. n. *faex, -cis*, yeast, feces; N.L. gen. n. *equifaecis*, from horse feces)

Cells of strain JHP9 are gram positive, non-motile, non-flagellate, and rod shaped, with a diameter of 0.8 × 1.8 µm. Colonies are cream colored, convex, smooth, glistening, and 0.5–1 mm in diameter on TSA incubated at 30°C for 7 d. It grows optimally at 30°C (growth range, 18–37°C), pH 7.0 (growth range, 6.0–9.0), and at a 0.5–1% (growth range 0–10%, wt/vol) salt concentration. Cell wall peptidoglycan of strain JHP9 contains amino acids, including *meso*-DAP, alanine, and glutamic acid. The major isoprenoid quinones and fatty acids were MK-7 and C_15:0_ anteiso and C_19:0_ cyclo *ω*8*c*, respectively. The main polar lipid profile comprised DPG, PG, and GL. The JHP9 strain is resistant to chloramphenicol and kanamycin. It hydrolyzes starch and DNA but not casein, cellulose, Tween 20, and Tween 80. The hydrolysis activity of *β*-glucosidase was positive but that of protease was negative. The strain is positive for catalase and negative for oxidase activity, indole formation, dissimilatory nitrate reduction, and urease activity. Fermentation activity was positive for d-ribose, d-galactose, d-glucose, d-mannose, methyl α-d-glucopyranoside, esculin, d-cellobiose, d-lactose, d-trehalose, d-raffinose, and glycogen. The genome of strain JHP9 was 3.08 Mbp in size, with a 71.1% G + C content.

Based on phenotypic, genotypic, and phylogenetic analyses, it was proposed that strain JHP9 represents a novel species with the name *B. equifaecis* sp. nov., with the type strain being JHP9 (=  KCTC 49746^T^ = JCM 35094^T^).

## MATERIALS AND METHODS

### Isolation and morphological characterization

The *B. equifaecis* JHP9 strain was obtained from the fecal samples of pasture breeding horses in Jeju Island, Republic of Korea (33°26'46.9"N, 126°33'50.7"E). In a 50-mL plastic tube, 10-g fecal samples were collected from the center of a horse fecal ball and transferred to the laboratory on ice. Nine samples (0.1 g each) were inoculated into 1-mL sterilized phosphate-buffered saline and diluted 10 times to a 10^−5^ dilution. Subcultures of 10^−3^, 10^−4^, and 10^−5^ dilutions were conducted on Lactobacilli MRS agar (BD Difco, Franklin Lakes, NJ, USA) by spreading 500 µL of each dilution and incubating them at 30°C for 72 h under ambient air conditions. After several subcultures, single colonies based on colony morphology and color were selected to inoculate onto fresh MRS agar at 30°C. Strain JHP9, isolated from the 10^−4^-diluted fecal sample, was inoculated onto MRS agar plates and incubated at 30°C for 48 h. The isolated strain was regularly cultured on TSA (Oxoid, Hampshire, UK) plates. Gram staining was performed using a Gram staining kit (BD Difco) according to the manufacturer’s instructions, and colony morphology was examined microscopically. The shape and size characteristics of the cells were confirmed using TEM (Tecnai G2 Spirit Twin, FEI; as installed at the Korea Basic Science Institute) after using 1% phosphotungstic acid for negative staining. Stock cultures were stored in 20% glycerol at −80°C until characterization.

### Phylogenetic analysis for strain identification

The isolated strain was identified using molecular phylogenetic analysis based on approximately 1,453 bp of the 16S rRNA gene sequences. For this purpose, the genomic DNA of *B. equifaecis* JHP9 was extracted using a genomic DNA extraction kit (Bioneer, Korea). The 16S rRNA gene was PCR amplified using universal primers 27F (5′-AGAGTTTGATCMTGGCTCAG-3′; *E. coli* position 8–27) and 1492R (5′-TACGGYTACCTTGTTACGACTT-3′; *E. coli* position 1492–1510) (59) and the purified PCR product sequenced by Macrogen Co. Ltd. (Republic of Korea) using an ABI 3730xl DNA Analyzer (Thermo Fisher Scientific, Waltham, MA, USA) with the BigDye Terminator v3.1 Cycle Sequencing Kit (Thermo Fisher Scientific) following the manufacturer’s protocols. Sequencing was conducted on each template using 518F (5′-CCAGCAGCCGCGGTAATACG-3′), 785F (5′-GGATTAGATACCCTGGTA-3′), 800R (5′-TACCAGGGTATCTAATCC-3′), and 907R (5′-CCGTCAATTCMTTTRAGTTT-3′) primers to cover the entire region of the 16S rRNA gene.

An almost-complete 16S rRNA gene sequence (approximately 1.5 kbp) was obtained by assembling the sequences using the BioEdit v.7.2.6 software (60) with the CAP contig assembly program and comparing it with the 16S rRNA gene sequence extracted from the whole genome sequence of strain JHP9 (see below). Then, the sequence was compared with the 16S rRNA gene sequences of related taxa obtained from the GenBank database and the EzBioCloud server (https://www.ezbiocloud.net). The 16S rRNA gene sequences of representative members of *Brachybacterium* and an outgroup bacterium, *Sanguibacter suarezii* NBRC16159^T^, which has <90% 16R rRNA gene similarity with strain JHP9, were aligned with that of the JHP9 strain using SILVA (http://www.arb-silva.de/aligner), where the secondary structure of the rRNA gene was considered (61). Phylogenetic tree construction was accomplished using maximum likelihood, neighbor-joining, and maximum parsimony methods implemented in the MEGA11 program (62). Kimura’s two-parameter model (63) was employed to calculate phylogenetic distances, and bootstrap analysis was conducted based on 1,000 resampled data sets.

Based on the 16S rRNA gene sequences, the strain shares a high sequence similarity (>97%) with *B. nesterenkovii* JCM11648^T^, *B. huguangmaarense* JCM30544^T^, *B. horti* KCTC39563^T^, and *B. rhamnosum* KCTC9917^T^. Therefore, these type strains were obtained from the Japanese Collection of Microbes (JCM) (JCM11648^T^ and JCM30544^T^) and the Korean Collection for Type Cultures (KCTC) (KCTC39563^T^ and KCTC9917^T^) to compare their phenotypic characteristics with those of the isolated strain.

### Physiological and biochemical characterization

The temperature, pH, and NaCl ranges for growth were determined in triplicate in tryptone soya broth (TSB; Oxoid). Growth temperature, NaCl concentration, and pH ranges were tested over the ranges of 4–45°C (4, 10, 15, 18, 25, 30, 37, 42, and 45°C) at pH 7 for temperature determination, 0–25% (wt/vol) NaCl (0, 0.5, 1, 2, 3, 6, 7, 8, 9, and 10%) at 30°C and pH 7 for NaCl concentration analysis, and pH 4–10 (at intervals of 0.5 pH units) for adjusted final pH using NaOH (1N) and HCl (1N) at 30°C. Four different buffers were used in the pH response analysis (final concentration, 10 mM): homopiperazine-1,4-bis (2-ethanesulfonic acid) (pH 4.0–5.0), 2-(N-morpholino)ethanesulfonic acid (pH 5.0–6.5), 1,3-bis[tris(hydroxymethyl)methylamino]propane (bispropane, pH 7.0–8.5), and 3-(cyclohexylamino)-1-propanesulfonic acid (pH 9.0–10.0). Bacterial growth was evaluated by measuring culture absorbance at 600 nm using a DS-11 spectrophotometer (DeNovix, Wilmington, DE, USA) at the Bio-Health Materials Core-Facility (Jeju National University, Republic of Korea) after incubation for 24 h, 48 h, and a week at different temperatures, salinities, and pH conditions.

Catalase activity was determined by observing bubble production in 3% (wt/vol) hydrogen peroxide solution (64), and oxidase activity was confirmed by observing color transitions to a deep purple or blue after adding drops of 1% (wt/vol) tetramethyl-*p*-phenylenediamine solution (bioMérieux, France) (65). Bacterial enzyme activities were examined using API ZYM and API 20 NE test strips (bioMérieux). API 20 NE test strips were also used to assess the ability of bacteria to assimilate carbohydrates. The bacterial ability to ferment and produce acid from various carbohydrates was tested with API 50 CHL test strips (bioMérieux) using API 50 CHB/E media with mineral oil under anaerobic conditions in an anaerobic chamber (Coy Laboratory Products, Grass Lake, MI, USA), according to a previously described method (66). Starch degradation and Tween 20 and Tween 80 hydrolysis were tested as previously described (67, 68). DNA hydrolysis was determined by culturing the cells on DNase Test Agar containing methyl green (BD Difco). A zone of clearing around the bacterial colonies indicated positive activity.

Additionally, six sugar sources (glucose, sucrose, galactose, arabinose, lactose, and mannose) were used at a ratio of 1% to determine fermentation activity in the strains JHP9, *B. nesterenkovii* JCM11648^T^, and *B*. *huguangmaarense* JCM30544^T^, along with two control LAB strains of *L. delbrueckii* subsp. *bulgaricus* KCTC3769^T^, under anaerobic conditions (see above). Briefly, 0.001% TSB media were prepared for *Brachybacterium* and LAB strains using phenol red and minimal media, called artificial freshwater medium (AFM) (15). Subsequently, bacteria were inoculated into each medium separately containing the six sugars. Incubation was performed at 30°C for 2 wk anaerobically, and after incubation, lactic acid production was observed by detecting a color change in the media. Alterations in pH and lactic acid production tended to cause color changes to the phenol red. Detailed methodology is provided in the Supplementary Information. In addition, lactic acid production was observed when 5-mL growth culture of strains JHP9, JCM 11648^T^, JCM 30544^T^, and KCTC 3769^T^ was supplemented with sugars under anaerobic conditions. Finally, the lactic acid concentration was measured with an EZ-Lactate Assay kit (DoGenBio, Korea) and a SpectraMax iD5 microplate reader (Molecular Devices, San Jose, CA, USA) at the Bio-Health Materials Core-Facility (Jeju National University) according to the manufacturer’s instructions.

The disk diffusion method was used to determine antibiotic susceptibility patterns of the bacteria (69). The following antibiotics were tested in the present study: ampicillin (10 µg per disk), streptomycin (10 µg per disk), chloramphenicol (30 µg per disk), ciprofloxacin (5 µg per disk), gentamicin (10 µg per disk), kanamycin (30 µg per disk), and tetracycline (30 µg per disk). These antibiotics were selected on the basis of their common use for therapeutic and prophylactic purposes in horse (https://www.woah.org/app/uploads/2021/03/oie-list-antimicrobials.pdf). Antibiotic susceptibility was defined based on breakpoints provided by the Clinical and Laboratory Standards Institute guidelines.

### Chemotaxonomic analyses

Chemotaxonomic analyses were performed for peptidoglycan, fatty acids, polar lipids, and menaquinone after culturing cells for 48 h on TSA (1% [wt/vol] NaCl, pH 7) at 30°C. Briefly, cell wall peptidoglycan of strain JHP9 was hydrolyzed and extracted using 6 N HCl at 121°C for 15 min and analyzed on a cellulose TLC plate according to a previously described method (70). Cellular fatty acids were extracted from strains JHP9, JCM11648^T^, and JCM30544^T^ via gas chromatography (HP7890 GC-FID; Agilent Technologies, Santa Clara, CA, USA) according to protocols of the Sherlock Microbial Identification System (MIDI; version 6.1) and the TSBA6 database (71). Identification and quantification of the extracted fatty acids were performed using the Sherlock MIDI system and standard MIS library generation software (version 6.3; Microbial ID Inc., Newark, DE, USA). Polar lipids were extracted using a chloroform-methanol solution (2:1, vol/vol) according to a previously described method (72) and analyzed using two-dimensional TLC. Furthermore, aminolipids, phospholipids, and glycolipids were identified using ninhydrin, molybdenum blue spray reagent, and α-naphthol-sulfuric acid, respectively, by heating at 110°C for 15 min. The isoprenoid quinones of strain JHP9 were identified with chloroform: methanol (2:1, vol/vol) using high-performance liquid chromatography (HPLC 2487; Waters Corporation, Milford, MA, USA) with a reversed-phase column, as described previously (73, 74). Detailed chemotaxonomic analysis methodology is provided in the Supplementary Information.

### Genome sequencing and analyses

Genomic DNA was extracted from the isolated bacteria using a QIAamp DNA Mini Kit (Qiagen, Germany) according to the manufacturer’s instructions. We previously described the draft genome sequence of the novel JHP9 genome (75). After quantification, the genomic DNA was sequenced at Macrogen, Inc. (Seoul, South Korea), using the Illumina HiSeqX platform. Raw reads were verified for quality using FastP v0.23.1 (76), followed by assembly using Spades v1.22 (77) with the default parameters. The assembled genome was validated using a self-mapping strategy and BUSCO analysis (78). BUSCO analysis is performed to evaluate the genome assembly based on evolutionarily informed expectations of gene content. The filtered reads were aligned against the assembled genome, and their insert sizes were estimated for validation.

Genome annotation was performed with Prokka v1.13 (79) using default parameters and the Pfam database (80) with a BLAST identity >80%. In addition, CAZymes involved in the synthesis, metabolism, and recognition of complex carbohydrates were predicted using the CAZymes Analysis Toolkit (81) and dbCAN HMM version 4.0 (82), using the CAZy database. Secondary metabolite biosynthetic gene clusters were identified using antiSMASH 6.0 software (83) with strict detection. Genome sequences were further screened for ARGs using AMRFinderPlus (84) with default parameters. Potential virulence genes were identified using a virulence factor database (VFDB) with the VFDB core data set of proteins associated with experimentally verified virulence factors (85). MGEs in the genomes, including insertion sequence elements and transposons, were detected by using IseScan (86) and TEfinder (87), respectively. CRISPR systems in *Brachybacterium* genomes were identified using the CRISPR comparison toolkit (88), and prophages were identified with Phispy v4.2.19 (89) without HMM searches. Additionally, functional assignment through homology searching against the specialized Kyoto Encyclopedia of Genes and Genomes database (90) was performed to determine the metabolic potential of the *Brachybacterium* genomes. All available, nearly-complete genomes of the *Brachybacterium* genus were obtained from the NCBI genome database (http://www.ncbi.nlm.nih.gov/genome/) and used for comparative analyses.

Genome distinctiveness was evaluated using genome-relatedness parameters, including ANI, AAI, and *is*DDH. The ANI and AAI calculations were performed using FastANI (91) and CompareM (https://github.com/dparks1134/CompareM), respectively. The *is*DDH values were calculated with the Genome-to-Genome Distance Calculator version 2.1 (http://ggdc.dsmz.de/distcalc2.php), using the recommended BLAST+ alignment (17).

Six *Brachybacterium* genomes were obtained from the NCBI genome database for comparative analysis with *B. equifaecis* JHP9. These genomes were chosen based on their similarity to *B. equifaecis* JHP9, as determined through phylogenetic analysis using 16S rRNA gene sequences. Additionally, we considered genome completeness of over 90%, which was assessed using BUSCO analysis (Table S1). Moreover, 163 complete representative genomes within the order *Micrococcales*, to which *Brachybacterium* belongs, were downloaded from the NCBI genome database for the phylogenetic clustering of *B. equifaecis* JHP9 within this order (Supplementary File 1).

Pangenome analysis of the available *Brachybacterium* and JHP9 genomes was performed using the Bacterial Pan Genome Analysis tool (BPGA) version 1.0 (92) with sequence identity ≥50% and an E value ≤1.0 × 10^−5^. Core, accessory, and unique genes were classified into orthologous groups using the USEARCH clustering algorithm (93). The *Brachybacterium* core genome sequences obtained from BPGA analysis were aligned against the NCBI RefSeq database (94) using BLAST with an identity cutoff of 50% to determine the closely associated genomes within the *Actinomycetota* phylum. Then, the closely associated 163 complete genomes (Supplementary File 1) within the order *Micrococcales* of the phylum *Actinomycetota* were downloaded from NCBI genome downloading scripts (available at https://github.com/kblin/ncbi-genome-download). These and the JHP9 genomes were subjected to alignment-free composition vector-based phylogenetic analyses using the CVTree4 program (95) with K = 6, and the resulting phylogenetic tree visualized and edited using iTOL v.6.5.2 (96).

### Determining kinetic properties

Kinetic properties were quantified by measuring the glucose- and oxygen-dependent oxygen uptake in an MR system (Unisense, Denmark) equipped with a PA 2000 picoammeter and an OX-MR oxygen microsensor (500-µm-diameter tip; Unisense, Denmark), polarized continuously for at least 24 h before use (15). MR experiments were conducted with each *Brachybacterium* strain under the respective cultivation conditions. To concentrate the biomass for MR, actively growing cells were collected from 5-mL culture by centrifugation (8,000 × *g*, 10 min, 28°C). The concentrated biomass was washed twice with fresh AFM and resuspended in AFM for the MR experiments. The culture biomass was incubated in a water bath set to the experimental temperature prior to being transferred to a 2-mL glass MR chamber with a stir bar. All MR experiments were performed with 300 rpm stirring at 28°C. Small culture volumes were frequently used for glucose measurements during the experiment to confirm the stoichiometric conversion of oxygen in the glucose-dependent oxygen uptake experiments. The glucose concentration was measured using a Glucose Colorimetric Detection Kit (Thermo Fisher Scientific) according to the manufacturer’s instructions. For oxygen-dependent oxygen uptake, 2 mM glucose was added to the bacterial cell-suspended chamber. Cell abundance was determined via qPCR using the bacterial primers, 518F-786R, as described by Jung et al. (97). The cell numbers used for the MR experiments were ~1.7 × 10^8^ for strain JHP9, ~9.1 × 10^8^ for *B. nesterenkovii*, ~6.8 × 10^7^ for *B. huguangmaarense*, ~4.9 × 10^8^ for *B. horti*, and ~6.2 × 10^8^.

### Data and statistical analysis

Resulting data from the physiological and chemotaxonomic analyses were analyzed descriptively to compare the characteristics of strain JHP9 with those of the reference strains. Statistical analyses were performed using the R computing environment (http://www.R-project.org/) and SigmaPlot 11.0 (Systat Software Inc., San Jose, CA, USA). Differences between lactic acid production values were analyzed using one-way analysis of variance (ANOVA). The significance level (α) for the ANOVA was 0.05. Tukey’s comparison of means was used to identify the significant condition effects. The heatmaply package in R (https://cran.r-project.org/web/packages/heatmaply/index.html) was used to visualize genome similarity matrices.

## Supplementary Material

Reviewer comments

## Data Availability

The GenBank/EMBL/DDBJ accession number for the 16S rRNA gene is OL468816, whereas the NCBI GenBank accession number for the whole-genome sequence of JHP9 is JAKNCJ010000000. Strain JHP9 has been deposited in the KCTC (KCTC 49746) and the JCM (JCM 35094).

## SUPPLEMENTAL MATERIAL

Data Set S1 spectrum.05048-22-s0001.xlsx Genome information for phylogenetic analysis in Fig. 1.

Data Set S2 spectrum.05048-22-s0002.xlsx CRISPR-cas types, terminal oxidases, MGEs, CAZYmes, and stress and antibiotic resistance genes.

Text S1 spectrum.05048-22-s0003.pdf Supplemental methods, tables, and figures.

OPEN PEER REVIEW reviewer-comments.pdf An accounting of the reviewer comments and feedback.

ASM does not own the copyrights to Supplemental Material that may be linked to, or accessed through, an article. The authors have granted ASM a non-exclusive, world-wide license to publish the Supplemental Material files. Please contact the corresponding author directly for reuse.

## References

[1] Louca S , Mazel F , Doebeli M , Parfrey LW . 2019. A census-based estimate of earth’s bacterial and archaeal diversity. PLoS Biol 17:e3000106. doi:10.1371/journal.pbio.3000106 30716065 PMC6361415

[2] Kauter A , Epping L , Semmler T , Antao E-M , Kannapin D , Stoeckle SD , Gehlen H , Lübke-Becker A , Günther S , Wieler LH , Walther B . 2019. The gut microbiome of horses: current research on equine enteral microbiota and future perspectives. Anim Microbiome 1:14. doi:10.1186/s42523-019-0013-3 33499951 PMC7807895

[3] Wunderlich G , Bull M , Ross T , Rose M , Chapman B . 2023. Understanding the microbial fibre degrading communities & processes in the equine gut. Anim Microbiome 5:3. doi:10.1186/s42523-022-00224-6 36635784 PMC9837927

[4] Liu Y , Zhai L , Yao S , Cao Y , Cao Y , Zhang X , Su J , Ge Y , Zhao R , Cheng C . 2015. Brachybacterium hainanense sp. nov., isolated from noni (Morinda citrifolia L.) branch. Int J Syst Evol Microbiol 65:4196–4201. doi:10.1099/ijsem.0.000559 26311250

[5] Chang DH , Rhee MS , Kim BC . 2016. Dermabacter vaginalis sp. nov., isolated from human vaginal fluid. Int J Syst Evol Microbiol 66:1881–1886. doi:10.1099/ijsem.0.000960 26867728

[6] Park YK , Lee KM , Lee WK , Cho MJ , Lee HS , Cho YG , Lee YC , Lee WK , Seong WK , Hwang KJ . 2016. Dermabacter jinjuensis sp. nov., a novel species of the genus Dermabacter isolated from a clinical specimen. Int J Syst Evol Microbiol 66:2573–2577. doi:10.1099/ijsem.0.001092 27088668

[7] Tak EJ , Kim PS , Hyun D-W , Kim HS , Lee J-Y , Kang W , Sung H , Shin N-R , Kim M-S , Whon TW , Bae J-W . 2018. Phenotypic and genomic properties of Brachybacterium vulturis sp. nov. and Brachybacterium avium sp. nov. Front. Microbiol 9. doi:10.3389/fmicb.2018.01809 PMC6090031 30131788

[8] Mekhalif F , Tidjani Alou M , Zgheib R , Lo CI , Fournier PE , Raoult D , Lagier JC . 2019. Brachybacterium massiliense sp. nov., a new bacterium isolated from stool from a healthy senegalese child. New Microbes New Infect 31:100588. doi:10.1016/j.nmni.2019.100588 31463068 PMC6710231

[9] Tamai K , Akashi Y , Yoshimoto Y , Yaguchi Y , Takeuchi Y , Shiigai M , Igarashi J , Hirose Y , Suzuki H , Ohkusu K . 2018. First case of a bloodstream infection caused by the genus Brachybacterium. J Infect Chemother 24:998–1003. doi:10.1016/j.jiac.2018.06.005 30007866

[10] van Elsas JD , Semenov AV , Costa R , Trevors JT . 2011. Survival of Escherichia coli in the environment: fundamental and public health aspects. ISME J 5:173–183. doi:10.1038/ismej.2010.80 20574458 PMC3105702

[11] Hibbing ME , Fuqua C , Parsek MR , Peterson SB . 2010. Bacterial competition: surviving and thriving in the microbial jungle. Nat Rev Microbiol 8:15–25. doi:10.1038/nrmicro2259 19946288 PMC2879262

[12] Chou JH , Lin KY , Lin MC , Sheu SY , Wei YH , Arun AB , Young CC , Chen WM . 2007. Brachybacterium phenoliresistens sp. nov., isolated from oil-contaminated coastal sand. Int J Syst Evol Microbiol 57:2674–2679. doi:10.1099/ijs.0.65019-0 17978239

[13] Hlaing PPT , Junqueira ACM , Uchida A , Purbojati RW , Houghton JNI , Chénard C , Wong A , Clare ME , Kushwaha KK , Putra A , Kee C , Gaultier NE , Premkrishnan BNV , Heinle CE , Lim SBY , Vettah VK , Drautz-Moses DI , Schuster SC . 2019. Complete genome sequence of Brachybacterium sp. Strain SGAir0954, isolated from Singapore air. Microbiol Resour Announc 8:e00619-19. doi:10.1128/MRA.00619-19 31395638 PMC6687925

[14] Ming H , Cheng LJ , Yi BF , Xia TT , Niu MM , Zhao ZY , Liu BB , Nie GX , Cui CX . 2021. Brachybacterium subflavum sp. nov., a novel actinobacterium isolated from the foregut of grass carp. Int J Syst Evol Microbiol 71. doi:10.1099/ijsem.0.004839 34170217

[15] Jung M-Y , Sedlacek CJ , Kits KD , Mueller AJ , Rhee S-K , Hink L , Nicol GW , Bayer B , Lehtovirta-Morley L , Wright C , de la Torre JR , Herbold CW , Pjevac P , Daims H , Wagner M . 2022. Ammonia-oxidizing archaea possess a wide range of cellular ammonia affinities. ISME J 16:272–283. doi:10.1038/s41396-021-01064-z 34316016 PMC8692354

[16] Ciufo S , Kannan S , Sharma S , Badretdin A , Clark K , Turner S , Brover S , Schoch CL , Kimchi A , DiCuccio M . 2018. Using average nucleotide identity to improve taxonomic assignments in prokaryotic genomes at the NCBI. Int J Syst Evol Microbiol 68:2386–2392. 29792589 10.1099/ijsem.0.002809 PMC6978984

[17] Meier-Kolthoff JP , Auch AF , Klenk HP , Göker M . 2013. Genome sequence-based species delimitation with confidence intervals and improved distance functions. BMC Bioinform 14:60. doi:10.1186/1471-2105-14-60 PMC3665452 23432962

[18] Konstantinidis KT , Tiedje JM . 2005. Towards a genome-based taxonomy for prokaryotes. J Bacteriol 187:6258–6264. doi:10.1128/JB.187.18.6258-6264.2005 16159757 PMC1236649

[19] Medlar AJ , Törönen P , Holm L . 2018. AAI-profiler: fast proteome-wide exploratory analysis reveals taxonomic identity, misclassification and contamination. Nucleic Acids Res 46:W479–W485. doi:10.1093/nar/gky359 29762724 PMC6030964

[20] Gvozdyak OR , Nogina TM , Schumann P . 1992. Taxonomic study of the genus Brachybacterium: Brachybacterium nesterenkovii sp. nov. Int J Syst Bacteriol 42:74–78. doi:10.1099/00207713-42-1-74 1736971

[21] Leyn SA , Maezato Y , Romine MF , Rodionov DA . 2017. Genomic reconstruction of carbohydrate utilization capacities in microbial-mat derived consortia. Front Microbiol 8:1304. doi:10.3389/fmicb.2017.01304 28751880 PMC5507952

[22] Stolper DA , Revsbech NP , Canfield DE . 2010. Aerobic growth at nanomolar oxygen concentrations. Proc Natl Acad Sci U S A 107:18755–18760. doi:10.1073/pnas.1013435107 20974919 PMC2973883

[23] Bodelier PLE , Laanbroek HJ . 1997. Oxygen uptake kinetics of Pseudomonas chlororaphis grown in glucose- or glutamate-limited continuous cultures. Arch Microbiol 167:392–395. doi:10.1007/s002030050460

[24] Martens-Habbena W , Berube PM , Urakawa H , de la Torre JR , Stahl DA . 2009. Ammonia oxidation kinetics determine niche separation of nitrifying archaea and bacteria. Nature 461:976–979. doi:10.1038/nature08465 19794413

[25] Cox RP , Marling N . 1992. High-affinity oxygen uptake by Bifidobacterium bifidum. Antonie Van Leeuwenhoek 62:291–297. doi:10.1007/BF00572597 1285646

[26] Hardin G . 1960. The competitive exclusion principle. Science 131:1292–1297. doi:10.1126/science.131.3409.1292 14399717

[27] Abbott DW , van Bueren AL . 2014. Using structure to inform carbohydrate binding module function. Curr Opin Struct Biol 28:32–40. doi:10.1016/j.sbi.2014.07.004 25108190

[28] Munir RI , Schellenberg J , Henrissat B , Verbeke TJ , Sparling R , Levin DB . 2014. Comparative analysis of carbohydrate active enzymes in clostridium termitidis CT1112 reveals complex carbohydrate degradation ability. PLoS One 9:e104260. doi:10.1371/journal.pone.0104260 25101643 PMC4125193

[29] Yao T , Chen M-H , Lindemann SR . 2020. Structurally complex carbohydrates maintain diversity in gut-derived microbial consortia under high dilution pressure. FEMS Microbiol Ecol 96:fiaa158. doi:10.1093/femsec/fiaa158 32815998

[30] Singhvi M , Zendo T , Sonomoto K . 2018. Free lactic acid production under acidic conditions by lactic acid bacteria strains: challenges and future prospects. Appl Microbiol Biotechnol 102:5911–5924. doi:10.1007/s00253-018-9092-4 29804138

[31] George F , Daniel C , Thomas M , Singer E , Guilbaud A , Tessier FJ , Revol-Junelles AM , Borges F , Foligné B . 2018. Occurrence and dynamism of lactic acid bacteria in distinct ecological niches: a multifaceted functional health perspective. Front Microbiol 9:2899. doi:10.3389/fmicb.2018.02899 30538693 PMC6277688

[32] Abedi E , Hashemi SMB . 2020. Lactic acid production–producing microorganisms and substrates sources-state of art. Heliyon 6:e04974. doi:10.1016/j.heliyon.2020.e04974 33088933 PMC7566098

[33] Montero-Zamora J , Fernández-Fernández S , Redondo-Solano M , Mazón-Villegas B , Mora-Villalobos JA , Barboza N . 2022. Assessment of different lactic acid bacteria isolated from agro-industrial residues: first report of the potential role of Weissella soli for lactic acid production from milk whey. Appl Microbiol 2:626–635. doi:10.3390/applmicrobiol2030048

[34] Liu Y , Xie QY , Shi W , Li L , An JY , Zhao YM , Hong K . 2014. Brachybacterium huguangmaarense sp. nov., isolated from lake sediment. Int J Syst Evol Microbiol 64:1673–1678. doi:10.1099/ijs.0.052464-0 24532648

[35] Esposti MD . 2020. On the evolution of cytochrome oxidases consuming oxygen. Biochim Biophys Acta Bioenerg 1861:148304. doi:10.1016/j.bbabio.2020.148304 32890468

[36] Rice CW , Hempfling WP . 1978. Oxygen-limited continuous culture and respiratory energy conservation in Escherichia coli. J Bacteriol 134:115–124. doi:10.1128/jb.134.1.115-124.1978 25879 PMC222225

[37] D’Mello R , Hill S , Poole RK . 1995. The oxygen affinity of cytochrome bo' in Escherichia coli determined by the deoxygenation of oxyleghemoglobin and oxymyoglobin: km values for oxygen are in the submicromolar range. J Bacteriol 177:867–870. doi:10.1128/jb.177.3.867-870.1995 7836332 PMC176676

[38] Jahreis K , Pimentel-Schmitt EF , Brückner R , Titgemeyer F . 2008. Ins and outs of glucose transport systems in eubacteria. FEMS Microbiol Rev 32:891–907. doi:10.1111/j.1574-6976.2008.00125.x 18647176

[39] Button DK . 1991. Biochemical basis for whole-cell uptake kinetics: specific affinity, oligotrophic capacity, and the meaning of the michaelis constant. Appl Environ Microbiol 57:2033–2038. doi:10.1128/aem.57.7.2033-2038.1991 16348524 PMC183517

[40] Ishida Y , Imai I , Miyagaki T , Kadota H . 1982. Growth and uptake kinetics of a facultatively oligotrophic bacterium at low nutrient concentrations. Microb Ecol 8:23–32. doi:10.1007/BF02011458 24225695

[41] Martens-Habbena W , Stahl DA . 2011. Nitrogen metabolism and kinetics of ammonia-oxidizing archaea. Methods Enzymol 496:465–487. doi:10.1016/B978-0-12-386489-5.00019-1 21514476

[42] Corona F , Martinez JL . 2013. Phenotypic resistance to antibiotics. Antibiotics (Basel) 2:237–255. doi:10.3390/antibiotics2020237 27029301 PMC4790337

[43] Depardieu F , Podglajen I , Leclercq R , Collatz E , Courvalin P . 2007. Modes and modulations of antibiotic resistance gene expression. Clin Microbiol Rev 20:79–114. doi:10.1128/CMR.00015-06 17223624 PMC1797629

[44] Hughes D , Andersson DI . 2017. Environmental and genetic modulation of the phenotypic expression of antibiotic resistance. FEMS Microbiol Rev 41:374–391. doi:10.1093/femsre/fux004 28333270 PMC5435765

[45] Murata K , Ozawa K , Kawakami H , Mochizuki K , Ohkusu K . 2020. Brachybacterium paraconglomeratum endophthalmitis postcataract operation. Case Rep Ophthalmol Med 2020:1513069. doi:10.1155/2020/1513069 32231828 PMC7091523

[46] Tian M , He X , Feng Y , Wang W , Chen H , Gong M , Liu D , Clarke JL , van Eerde A . 2021. Pollution by antibiotics and antimicrobial resistance in livestock and poultry manure in China, and countermeasures. Antibiotics (Basel) 10:539. doi:10.3390/antibiotics10050539 34066587 PMC8148549

[47] Goldfarb T , Sberro H , Weinstock E , Cohen O , Doron S , Charpak-Amikam Y , Afik S , Ofir G , Sorek R . 2015. BREX is a novel phage resistance system widespread in microbial genomes. EMBO J 34:169–183. doi:10.15252/embj.201489455 25452498 PMC4337064

[48] Hille F , Charpentier E . 2016. CRISPR-Cas: biology, mechanisms and relevance. Philos Trans R Soc Lond B Biol Sci 371:20150496. doi:10.1098/rstb.2015.0496 27672148 PMC5052741

[49] Mat Razali N , Cheah BH , Nadarajah K . 2019. Transposable elements adaptive role in genome plasticity, pathogenicity and evolution in fungal phytopathogens. Int J Mol Sci 20:3597. doi:10.3390/ijms20143597 31340492 PMC6679389

[50] Fan C , Wu Y-H , Decker CM , Rohani R , Gesell Salazar M , Ye H , Cui Z , Schmidt F , Huang WE . 2019. Defensive function of transposable elements in bacteria. ACS Synth Biol 8:2141–2151. doi:10.1021/acssynbio.9b00218 31375026

[51] Farooq A , Kim J , Raza S , Jang J , Han D , Sadowsky MJ , Unno T . 2021. A hybrid DNA sequencing approach is needed to properly link genotype to phenotype in multi-drug resistant bacteria. Environ Pollut 289:117856. doi:10.1016/j.envpol.2021.117856 34330011

[52] Borisov VB , Gennis RB , Hemp J , Verkhovsky MI . 2011. The cytochrome bd respiratory oxygen reductases. Biochim Biophys Acta Bioenerg 1807:1398–1413. doi:10.1016/j.bbabio.2011.06.016 PMC3171616 21756872

[53] D’mello R , Hill S , Poole RK . 1996. The cytochrome bd quinol oxidase in Escherichia coli has an extremely high oxygen affinity and two oxygen-binding haems: implications for regulation of activity in vivo by oxygen inhibition. Microbiology (Reading) 142 (Pt 4):755–763. doi:10.1099/00221287-142-4-755 8936304

[54] Bottacini F , Milani C , Turroni F , Sánchez B , Foroni E , Duranti S , Serafini F , Viappiani A , Strati F , Ferrarini A , Delledonne M , Henrissat B , Coutinho P , Fitzgerald GF , Margolles A , van Sinderen D , Ventura M . 2012. Bifidobacterium asteroides PRL2011 genome analysis reveals clues for colonization of the insect gut. PLoS One 7:e44229. doi:10.1371/journal.pone.0044229 23028506 PMC3447821

[55] Gong X , Garcia-Robledo E , Schramm A , Revsbech NP . 2015. Respiratory kinetics of marine bacteria exposed to decreasing oxygen concentrations. Appl Environ Microbiol 82:1412–1422. doi:10.1128/AEM.03669-15 26682857 PMC4771329

[56] Passalacqua KD , Charbonneau M-E , O’Riordan MXD . 2016. Bacterial metabolism shapes the host-pathogen interface. Microbiol Spectr 4. doi:10.1128/microbiolspec.VMBF-0027-2015 PMC4922512 27337445

[57] Woodcock DJ , Krusche P , Strachan NJC , Forbes KJ , Cohan FM , Méric G , Sheppard SK . 2017. Genomic plasticity and rapid host switching can promote the evolution of generalism: a case study in the zoonotic pathogen Campylobacter. Sci Rep 7:9650. doi:10.1038/s41598-017-09483-9 28851932 PMC5575054

[58] Hassan L . 2014. Emerging Zoonoses in domesticated livestock of Southeast Asia. 2nd ed. Elsvier, Amsterdam, AM.

[59] Weisburg WG , Barns SM , Pelletier DA , Lane DJ . 1991. 16S ribosomal DNA amplification for phylogenetic study. J Bacteriol 173:697–703. doi:10.1128/jb.173.2.697-703.1991 1987160 PMC207061

[60] Alzohairy A . 2011. BioEdit: an important software for molecular biology. GERF bull biosci 2:60–61.

[61] Pruesse E , Quast C , Knittel K , Fuchs BM , Ludwig W , Peplies J , Glöckner FO . 2007. SILVA: a comprehensive online resource for quality checked and aligned ribosomal RNA sequence data compatible with ARB. Nucleic Acids Res 35:7188–7196. doi:10.1093/nar/gkm864 17947321 PMC2175337

[62] Tamura K , Stecher G , Kumar S . 2021. MEGA11: molecular evolutionary genetics analysis version 11. Mol Biol Evol 38:3022–3027. doi:10.1093/molbev/msab120 33892491 PMC8233496

[63] Kimura M . 1980. A simple method for estimating evolutionary rates of base substitutions through comparative studies of nucleotide sequences. J Mol Evol 16:111–120. doi:10.1007/BF01731581 7463489

[64] Taylor WI , Achanzar D . 1972. Catalase test as an aid to the identification of enterobacteriaceae . Appl Microbiol 24:58–61. doi:10.1128/am.24.1.58-61.1972 4560474 PMC380547

[65] Tarrand JJ , Gröschel DH . 1982. Rapid, modified oxidase test for oxidase-variable bacterial isolates. J Clin Microbiol 16:772–774. doi:10.1128/jcm.16.4.772-774.1982 7153330 PMC272470

[66] Hanson A . 2008. Oxidative-Fermentative test protocol. ASM press, Washington, DC.

[67] Gordon RE , Smith MM . 1953. Rapidly growing, acid fast bacteria. I. species' descriptions of Mycobacterium phlei lehmann and neumann and Mycobacterium smegmatis (trevisan) lehmann and neumann. J Bacteriol 66:41–48. doi:10.1128/jb.66.1.41-48.1953 13069464 PMC357089

[68] Holding A , Collee J . 1971. Chapter I routine biochemical tests, p 1–32. In Methods in microbiology. Elsevier, Amsterdam, AM.

[69] de Fátima Silva Lopes M , Ribeiro T , Abrantes M , Figueiredo Marques JJ , Tenreiro R , Crespo MTB . 2005. Antimicrobial resistance profiles of dairy and clinical isolates and type strains of enterococci. Int J Food Microbiol 103:191–198. doi:10.1016/j.ijfoodmicro.2004.12.025 16083821

[70] Cummins CS , Harris H . 1956. The chemical composition of the cell wall in some gram-positive bacteria and its possible value as a taxonomic character. J Gen Microbiol 14:583–600. doi:10.1099/00221287-14-3-583 13346020

[71] Sasser M . 1990. Identification of bacteria by gas chromatography of cellular fatty acids, In MIDI technical NOTE 101. MIDI inc, Newark, DE.

[72] Schleifer K , Seidl P , Goodfellow M , Minnikin D . 1985. Chemical methods in bacterial systematics. The Society for Applied Bacteriology, London.

[73] Hiraishi A , Ueda Y , Ishihara J , Mori T . 1996. Comparative lipoquinone analysis of influent sewage and activated sludge by high-performance liquid chromatography and photodiode array detection. J Gen Appl Microbiol 42:457–469. doi:10.2323/jgam.42.457

[74] Collins MD , Jones D . 1981. Distribution of isoprenoid quinone structural types in bacteria and their taxonomic implication. Microbiol Rev 45:316–354. doi:10.1128/mr.45.2.316-354.1981 7022156 PMC281511

[75] Lee M , Farooq A , Jung M-Y , Kim S-J . 2022. Draft genome sequence of Brachybacterium sp. JHP9 isolated from horse feces in Jeju Island. Korean J Microbiol 58:102–104.

[76] Chen S , Zhou Y , Chen Y , Gu J . 2018. Fastp: an ultra-fast all-in-one FASTQ preprocessor. Bioinformatics 34:i884–i890. doi:10.1093/bioinformatics/bty560 30423086 PMC6129281

[77] Prjibelski A , Antipov D , Meleshko D , Lapidus A , Korobeynikov A . 2020. Using spades de novo assembler. Curr Protoc Bioinformatics 70:e102. doi:10.1002/cpbi.102 32559359

[78] Simão FA , Waterhouse RM , Ioannidis P , Kriventseva EV , Zdobnov EM . 2015. BUSCO: assessing genome assembly and annotation completeness with single-copy orthologs. Bioinformatics 31:3210–3212. doi:10.1093/bioinformatics/btv351 26059717

[79] Seemann T . 2014. Prokka: rapid prokaryotic genome annotation. Bioinformatics 30:2068–2069. doi:10.1093/bioinformatics/btu153 24642063

[80] Mistry J , Chuguransky S , Williams L , Qureshi M , Salazar GA , Sonnhammer ELL , Tosatto SCE , Paladin L , Raj S , Richardson LJ , Finn RD , Bateman A . 2021. Pfam: the protein families database in 2021. Nucleic Acids Res 49:D412–D419. doi:10.1093/nar/gkaa913 33125078 PMC7779014

[81] Park BH , Karpinets TV , Syed MH , Leuze MR , Uberbacher EC . 2010. CAZymes analysis toolkit (CAT): web service for searching and analyzing carbohydrate-active enzymes in a newly sequenced organism using CAZy database. Glycobiology 20:1574–1584. doi:10.1093/glycob/cwq106 20696711

[82] Yin Y , Mao X , Yang J , Chen X , Mao F , Xu Y . 2012. dbCAN: a web resource for automated carbohydrate-active enzyme annotation. Nucleic Acids Res 40:W445–W451. doi:10.1093/nar/gks479 22645317 PMC3394287

[83] Blin K , Shaw S , Kloosterman AM , Charlop-Powers Z , van Wezel GP , Medema MH , Weber T . 2021. antiSMASH 6.0: improving cluster detection and comparison capabilities. Nucleic Acids Res 49:W29–W35. doi:10.1093/nar/gkab335 33978755 PMC8262755

[84] Feldgarden M , Brover V , Haft DH , Prasad AB , Slotta DJ , Tolstoy I , Tyson GH , Zhao S , Hsu C-H , McDermott PF , Tadesse DA , Morales C , Simmons M , Tillman G , Wasilenko J , Folster JP , Klimke W . 2019. Validating the AMRFinder tool and resistance gene database by using antimicrobial resistance genotype-phenotype correlations in a collection of isolates. Antimicrob Agents Chemother 63:e00483-19. doi:10.1128/AAC.00483-19 31427293 PMC6811410

[85] Chen L , Yang J , Yu J , Yao Z , Sun L , Shen Y , Jin Q . 2005. VFDB: a reference database for bacterial virulence factors. Nucleic Acids Res 33:D325–D328. doi:10.1093/nar/gki008 15608208 PMC539962

[86] Xie Z , Tang H , Hancock J . 2017. ISEScan: automated identification of insertion sequence elements in prokaryotic genomes. Bioinformatics 33:3340–3347. doi:10.1093/bioinformatics/btx433 29077810

[87] Sohrab V , López-Díaz C , Di Pietro A , Ma L-J , Ayhan DH . 2021. TEfinder: a bioinformatics pipeline for detecting new transposable element insertion events in next-generation sequencing data. Genes (Basel) 12:224. doi:10.3390/genes12020224 33557410 PMC7914406

[88] Collins AJ , Whitaker RJ . 2022. CRISPR comparison toolkit (CCTK): rapid identification, visualization, and analysis of CRISPR array diversity. bioRxiv. doi:10.1101/2022.07.31.502198 PMC10457644 37459160

[89] Akhter S , Aziz RK , Edwards RA . 2012. Phispy: a novel algorithm for finding prophages in bacterial genomes that combines similarity- and composition-based strategies. Nucleic Acids Res 40:e126. doi:10.1093/nar/gks406 22584627 PMC3439882

[90] Kanehisa M , Furumichi M , Tanabe M , Sato Y , Morishima K . 2017. KEGG: new perspectives on genomes, pathways, diseases and drugs. Nucleic Acids Res 45:D353–D361. doi:10.1093/nar/gkw1092 27899662 PMC5210567

[91] Jain C , Rodriguez-R LM , Phillippy AM , Konstantinidis KT , Aluru S . 2018. High throughput ANI analysis of 90K prokaryotic genomes reveals clear species boundaries. Nat Commun 9:5114. doi:10.1038/s41467-018-07641-9 30504855 PMC6269478

[92] Chaudhari NM , Gupta VK , Dutta C . 2016. BPGA- an ultra-fast pan-genome analysis pipeline. Sci Rep 6:24373. doi:10.1038/srep24373 27071527 PMC4829868

[93] Edgar RC . 2010. Search and clustering orders of magnitude faster than BLAST. Bioinformatics 26:2460–2461. doi:10.1093/bioinformatics/btq461 20709691

[94] Pruitt KD , Tatusova T , Maglott DR . 2005. NCBI reference sequence (RefSeq): a curated non-redundant sequence database of genomes, transcripts and proteins. Nucleic Acids Res 33:D501–D504. doi:10.1093/nar/gki025 15608248 PMC539979

[95] Xu Z , Hao B . 2009. CVTree update: a newly designed phylogenetic study platform using composition vectors and whole genomes. Nucleic Acids Res 37:W174–W178. doi:10.1093/nar/gkp278 19398429 PMC2703908

[96] Letunic I , Bork P . 2021. Interactive tree of life (iTOL) v5: an online tool for phylogenetic tree display and annotation. Nucleic Acids Res 49:W293–W296. doi:10.1093/nar/gkab301 33885785 PMC8265157

[97] Jung M-Y , Well R , Min D , Giesemann A , Park S-J , Kim J-G , Kim S-J , Rhee S-K . 2014. Isotopic signatures of N_2_O produced by ammonia-oxidizing archaea from soils. ISME J 8:1115–1125. doi:10.1038/ismej.2013.205 24225887 PMC3996685

