# Supplementary material for: Kinetic, genomic, and physiological analysis reveals diversity in the ecological adaptation and metabolic potential of Brachybacterium equifaecis sp. nov. isolated from horse feces
Source: Microbiol Spectr. 2023 Sep 14;11(5):e05048-22. doi: 10.1128/spectrum.05048-22 (PMC10581053; doi:10.1128/spectrum.05048-22)

Supplementary information

Kinetic, Genomic, and Physiological Analysis Reveals Diversity in the Ecological Adaptation and Metabolic Potential of *Brachybacterium equifaecis* sp. nov. Isolated from Horse Feces

Running Title: Ecological and metabolic diversity of strain JHP9

Adeel Farooq[a*], Myunglip Lee[b*], Saem Han[c*], Gi-Yong Jung[d,e], So-Jeong Kim[d], Man-Young Jung[c,f#]

[a]Research Institute for Basic Sciences (RIBS), Jeju National University, 102 Jejudaehak-ro, Jeju, 63243, Republic of Korea

[b]Department of Marine Life Science, Jeju National University, 102 Jejudaehak-ro, Jeju 63243, Republic of Korea

[c]Interdisciplinary Graduate Programme in Advance Convergence Technology and Science, Jeju National University, 102 Jejudaehak-ro, Jeju 63243, Republic of Korea

[d]Mineral Resources Research Division, Korea Institute of Geoscience and Mineral Resources, Daejeon, 34132, Republic of Korea

[e]Department of Biological Sciences and Biotechnology, Chungbuk National University, Chungdae-ro 1, Seowon-Gu, Cheongju, Chungbuk 28644, Republic of Korea

[f]Department of Science Education, Jeju National University, 102 Jejudaehak-ro, Jeju 63243, Republic of Korea

[*]Adeel Farooq, Myunglip Lee, and Saem Han contributed equally to this work.

[#]To whom correspondence should be addressed.

E-mail: myjung@jejunu.ac.kr

Tel: +82-64-754-3282

MATERIAL and METHODS

Chemotaxonomic analyses

Chemotaxonomic analyses were performed for peptidoglycan, fatty acids, polar lipids, and menaquinone by culturing cells for 48 h on TSA [1% (w/v) NaCl, pH 7] at 30 °C. The cell wall peptidoglycan was hydrolyzed and extracted using 6 N HCl at 121 °C for 15 min and analyzed onto a cellulose thin layer chromatography (TLC) plate according to the method (1). Cellular fatty acids of the strain JHP9 and two other closely related strains were extracted according to MIDI. The fatty acids were extracted by gas chromatograph (HP7890 GC-FID; Agilent Technologies, Santa Clara, CA, USA) and the TSBA6 database of the Microbial Identification System (2). The identification and quantification of the fatty acids were performed using the Sherlock Microbial Identification System and the standard MIS Library Generation Software (VERSION 6.3, Microbial ID Inc., Newark, DE, USA). The polar lipids of cells were extracted using chloroform-methanol solution (2:1, v/v) according to a previously described method (3) and analyzed using two-dimensional TLC as described earlier (4). The separation of polar lipid was determined by two-dimensional TLC for first-dimensional TLC using chloroform-methanol-water (65:25:4, v/v) and second-dimensional TLC using chloroform-acetic acid-methanol-water (80:15:12:4, v/v). Separated total polar lipid spots on the TLC plates (Silica gel 60 F254, 25 Aluminium sheets 20*20c, Merck) were detected using 10 % phosphomolybdic acid hydrate on heating at 180 °C for 15min. Aminolipids, phospholipids, and glycolipids were determined using ninhydrin, molybdenum blue spray reagent, and α-naphthol-sulphuric acid, respectively, on heating at 110 °C for 15 min. Isoprenoid quinones of strain JHP9 extracted by chloroform: methanol (2: 1, v/v) were determined using high-performance liquid chromatography (HPLC 2487, Waters, USA) using the reversed-phase column as described previously (5, 6).

Lactic acid product of Fermentation experiment

Sugar sources of five types were used glucose, sucrose, galactose, arabinose, and lactose, according to the method (7). Fermentation experiment was compared using strain JHP9, *Br. nesterenkovii* JCM 11648^T, *Br. huguangmaarense* JCM 30544^T, and *Lactobacillus delbrueckii* subsp. *Bulgaricus* KCTC 3769^T under anaerobic conditions. The medium for the genus *Brachybacterium* was used by mixing 0.001% of tryptone soya broth (TSB) and 0.002% of phenol red with an AFM (artificial freshwater medium). AFM was prepared by adding 0.2 g of KH_2PO_4, 0.1 g of $CaCl_2·2H_2O$, 0.5 g of KCl, 0.4 g of $MgCl_2·6H_2O$, and 1 g of NaCl to 1 L of total volume. After sterilization of AFM, additionally, 10 ml of sterilized NH_4Cl (1 M), 1 ml of FeNa-EDTA (1000×), 2 ml of $NaHCO_3$ (1 M), 10 ml of HEPES buffer (pH 7.5, 1 M), 1ml of Trace Element (1000×), and 1ml of vitamin solution (1000×), was added to prepare it (8). After that, six sterilized sugar sources were added, 1% (w/v) each, and the pH of the medium was finally adjusted to 7.4 using sterilized NaOH (1 N) and HCl (1 N). The inoculated strains were prepared at AFM to have OD600 nm=0.1 and washed twice (10000 rpm, 10 min) using PBS (Phosphate Buffered Saline). In the experiment, five types of mediums with different sugar sources at 10 ml were inoculated to 50 µl of the prepared bacteria. These bacteria were incubated at 7 days and 30 ºC in an anaerobic chamber (Coy, USA) for an anaerobic condition. As the negative control, in 2 groups, the strains were inoculated with AFM without adding a sugar source, and the strains were not inoculated with AFM with added sugar source. After incubation, the color change of phenol red by pH and lactic acid quantity was determined to be OD550 nm and OD570 nm, respectively, using SpectraMax iD5 (Molecular Devices, USA) in Bio-Health Materials Core-Facility, Jeju National University.

Supplementary Table S1. Genome characteristics of novel *Brachybacterium equifaecis* JHP9 in comparison with the neighboring strains.

Genomes	Genome Length (bp)	No. of Contigs	G+C Contents	N50 (bp)	L50	Coding Sequences	RNAs	Genome Completeness (Single copy complete BUSCOs; %)
B. equifaecis	3082019	23	71.1	754379	2	2720	57	94.8
B. nestrenkovii	3021972	119	72.4	50997	17	2842	52	95.3
B. squillarum	3191479	8	72.8	390377	2	2956	56	93.2
B. muris	3647194	1	69.7	3647194	1	3397	55	94
B. sacelli	4869106	2	70.3	3140405	1	4575	83	91.7
B. vulturis	3796663	1	70.8	3796663	1	3468	57	96.1
B. phenoliresistens	4188165	35	73.2	167341	7	3795	56	92.6

Supplementary Table S2. API (ZYM, 20NE, 50CHL) tests determining enzyme activity, assimilation, and acid production for the strain JHP9 and its closely associated *Brachybacterium* species. Strains 1, *Brachybacterium equifaecis* JHP9; 2, *Brachybacterium nesterenkovii* JCM11648^T; 3, *Brachybacterium huguangmaarense* JCM30544^T. (+, positive; W, weak; -, negative)

API ZYM test result was all positive for the enzymatic activity of esterase lipase (C8), leucine arylamidase, valine arylamidase, cystine arylamidase, acid phosphatase, *α*-galactosidase, *α*-glucosidase, and *N*–acetyl-*β*-glucosaminidase but negative of *α*-chymotrypsin, *α*–mannosidase, and *α*-fucosidase.

API 20NE test result was all positive for *β*-glucosidase (esculin hydrolysis), *β*-galactosidase (PNPG), D-glucose, *N*-Acetyl-D-glucosamine, and gluconate but the negative for nitrate reduction to nitrite and dinitrogen, indole production, glucose acidification, arginine dihydrolase, urease, protease (gelatin hydrolysis), L-arabinose, and caprate.

API 50CHL test result was all positive for D-ribose, D-galactose, D-glucose, D-mannose, and esculin but negative for erythritol, D-arabinose, L-xylose, D-adonitol, methyl-*β*-D-xylopyanoside, L-sorbose, L-rhamnose, dulcitol, inositol, D-sorbitol, methyl- *α* -D-mannopyranoside, D-melibiose, inulin, D-melezitose, xylitol, D-turanose, D-lyxose, D-tagatose, D-fucose, L-fucose, D-arabitol, L-arabitol, potassium gluconate, potassium 2-ketogluconate, and potassium 5-ketogluconate.

**Test**	**1**	**2**	**3**
**Enzyme activity**			
Alkaline phosphatase	w	-	-
Esterase (C4)	-	+	+
Lipase (C14)	-	+	-
Naphtol-AS-BI-phosphohydrolase	+	-	+
β-galactosidase	-	+	+
β-glucuronidase	+	-	-
β-glucosidase	-	+	+
**Assimilation**			
D-Mannose	-	-	+
D-Mannitol	-	-	w
D-Maltose	+	+	-
Adipate	-	+	+
Malate	+	-	w
Citrate	-	+	+
Phenyl-acetate	-	-	w
**Acid production and fermentation**			
Glycerol	-	+	+

L-Arabinose	-	+	-
D-Xylose	-	+	-
D-Fructose	-	+	+
D-Mannitol	-	-	+
Methyl-a-D-glucopyranoside	+	-	-
N-acetylglucosamine	-	+	-
Amygdalin	-	+	-
Arbutin	-	+	+
Salcin	-	+	+
D-Cellobiose	+	-	+
D-Maltose	-	+	+
D-Lactose	+	+	-
D-Saccharose	-	+	+
D-Trehalose	+	-	+
D-Raffinose	+	-	+
Amidon (starch)	-	+	+
Glycogen	+	-	+
Gentiobiose	-	+	-

Supplementary Table S3. Fatty acid composition of the **1**, *Brachybacterium equifaecis* JHP9; **2**, *Brachybacterium nesterenkovii* JCM11648^{T}; **3**, *Brachybacterium huguangmaarense* JCM30544^{T}. Fatty acids amounting to <1% of the total fatty acids in all strains are not listed. Major fatty acid components (>5.0 %) are highlighted in bold. TR, Trace (<1%); -, not detected.

Fatty acid	1	2	3
**Saturated**			
$C_{10:0}$	TR	-	TR
$C_{12:0}$	2.13	TR	4.12
$C_{14:0}$	1.61	1.52	3.00
$C_{16:0}$	1.81	1.27	3.84
$C_{18:0}$	TR	-	TR
**Unsaturated**			
$C_{18:1}$ *ω9c*	TR	-	TR
**Branched-chain fatty acid**			
$C_{14:0}$ iso	1.85	2.00	TR
$C_{15:0}$ iso	2.10	**5.58**	1.54
$C_{16:0}$ iso	**6.69**	1.22	3.59
$C_{17:0}$ iso	TR	-	TR
$C_{18:0}$ iso	TR	-	-
$C_{13:0}$ anteiso	-	TR	-
$C_{15:0}$ anteiso	**55.13**	**69.79**	**62.41**
$C_{17:0}$ anteiso	**8.81**	**8.95**	**12.45**
$C_{19:0}$ anteiso	TR	-	-
**CYCLO**			
$C_{19:0}$ cyclo *ω8c*	**11.49**	**7.84**	3.31
**Other**			
$C_{16:0}$ N alcohol	TR	-	1.09
**Summed feature***			
**3**; $C_{16:1}$ ω7c/$C_{16:1}$ *ω6c*	TR	-	-
**4**; $C_{17:1}$ iso I/$C_{17:1}$ anteiso B	-	-	1.14
**8**; $C_{18:1}$ ω7c/$C_{18:1}$ *ω6c*	4.41	1.15	TR

*****Summed features represent groups of two fatty acids that could not be separated by GLC with the MIDI system)

Supplementary Table S4. Antibiotics susceptibility test for the *Brachybacterium equifaecis* JHP9, *Brachybacterium nesterenkovii* JCM11648 ^{T}, and *Brachybacterium huguangmaarense* JCM30544^{T}.

**Antibiotics (μg)**	***Brachybacterium equifaecis* JHP9**	***Brachybacterium nesterenkovii* JCM11648^{T}**	***Brachybacterium huguangmaarense* JCM30544^{T}**
Ampicillin (10)	S	S	S
Chloramphenicol (30)	R	S	S
Gentamicin (10)	S	S	S
Kanamycin (30)	R	R	R
Streptomycin (10)	S	S	S
Tetracycline (30)	S	S	S

R, Resistant; S, Susceptible.

Supplementary References

1. Cummins CS, Harris H. 1956. The chemical composition of the cell wall in some gram-positive bacteria and its possible value as a taxonomic character. *J Gen Microbiol* 14:583-600.

2. Sasser M. 1990. Identification of bacteria by gas chromatography of cellular fatty acids. MIDI technical note 101. Newark, DE: MIDI inc.

3. Minnikin DE, O'Donnell AG, Goodfellow M, Alderson G, Athalye M, Schaal A, Parlett JH. 1984. An integrated procedure for the extraction of bacterial isoprenoid quinones and polar lipids. *J Microbiol Methods* 2:233-241.

4. Hoang VA, Kim YJ, Nguyen NL, Yang DC. 2014. *Brachybacterium ginsengisoli* sp. nov., isolated from soil of a ginseng field. *Int J Syst Evol Microbiol* 64:3063-3068.

5. Collins MD, Jones D. 1981. Distribution of isoprenoid quinone structural types in bacteria and their taxonomic implication. *Microbiol Rev* 45:316-54.

6. HIRAISHI A, UEDA Y, ISHIHARA J, MORI T. 1996. Comparative lipoquinone analysis of influent sewage and activated sludge by high-performance liquid chromatography and photodiode array detection. *J Gen Appl Microbiol* 42:457-469.

7. Hanson A. 2008. Oxidative-fermentative test protocol. ASM press, Washington, DC.

8. Jung M-Y, Sedlacek CJ, Kits KD, Mueller AJ, Rhee S-K, Hink L, Nicol GW, Bayer B, Lehtovirta-Morley L, Wright C, de la Torre JR, Herbold CW, Pjevac P, Daims H, Wagner M. 2022. Ammonia-oxidizing archaea possess a wide range of cellular ammonia affinities. *ISME J* 16:272-283.

Supplementary Fig. S1. TEM analysis of cells from exponential growth stage showing a cell with scale bar = 500 nm.

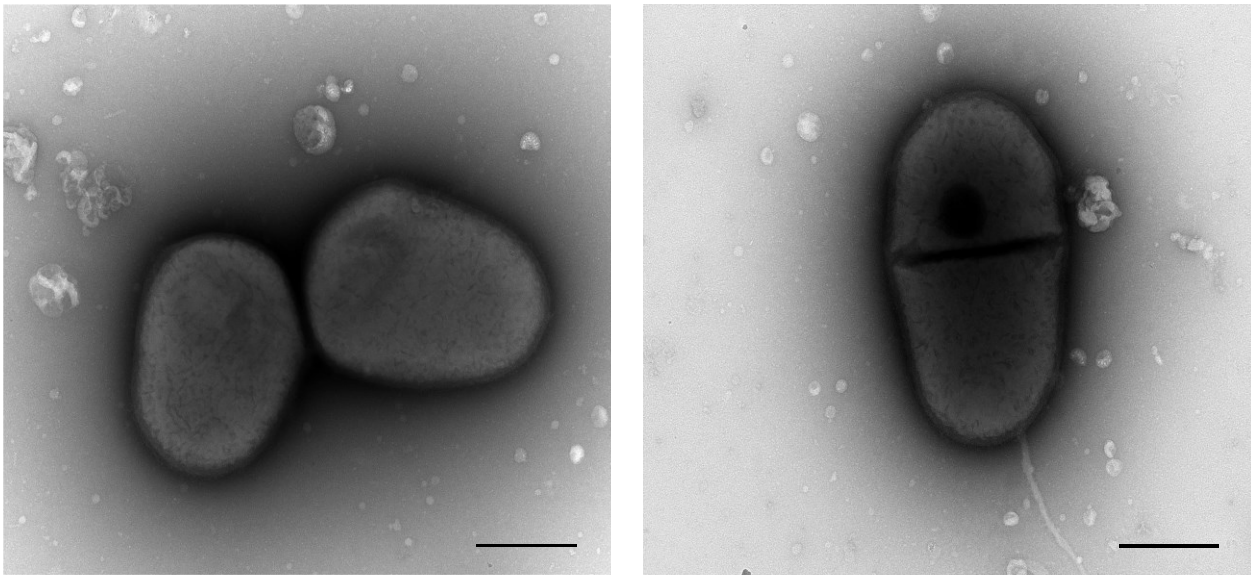

Supplementary Fig. S2. Chromatographic representation of major polar lipids of the strain JHP9. Major polar lipids were including diphosphatidylglycerol (DPG), phosphatidylglycerol (PG), and glycerolipids (GL).

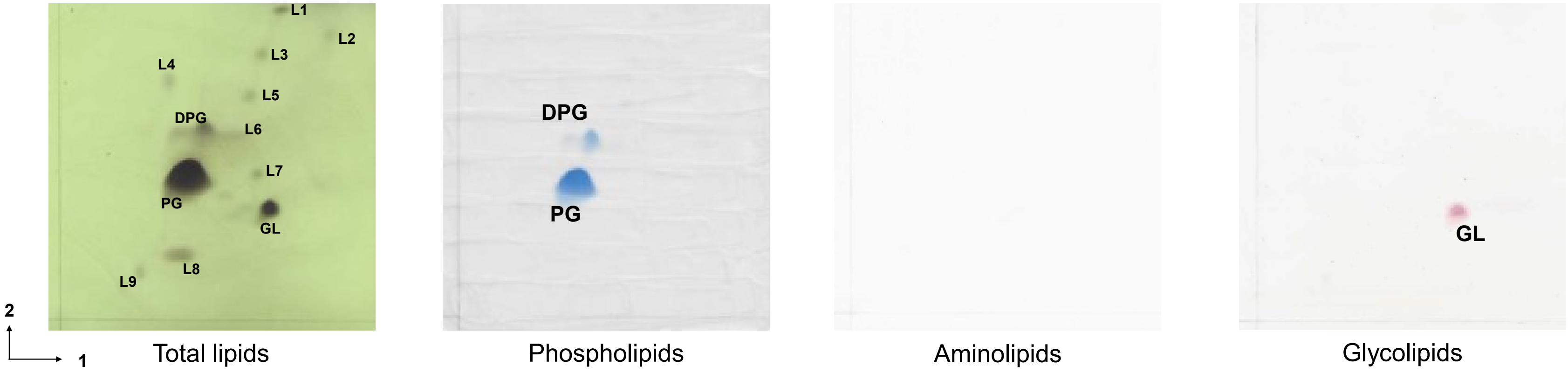

Supplementary Fig. S3. Schematic metabolic potential of *Brachybacterium* spp. (n=7), including the novel strain JHP9. Metabolism pathways were reconstructed based on KEGG annotation. The green dots indicate the presence, and the black dots indicate the absence of respective proteins across 4 or more genomes.

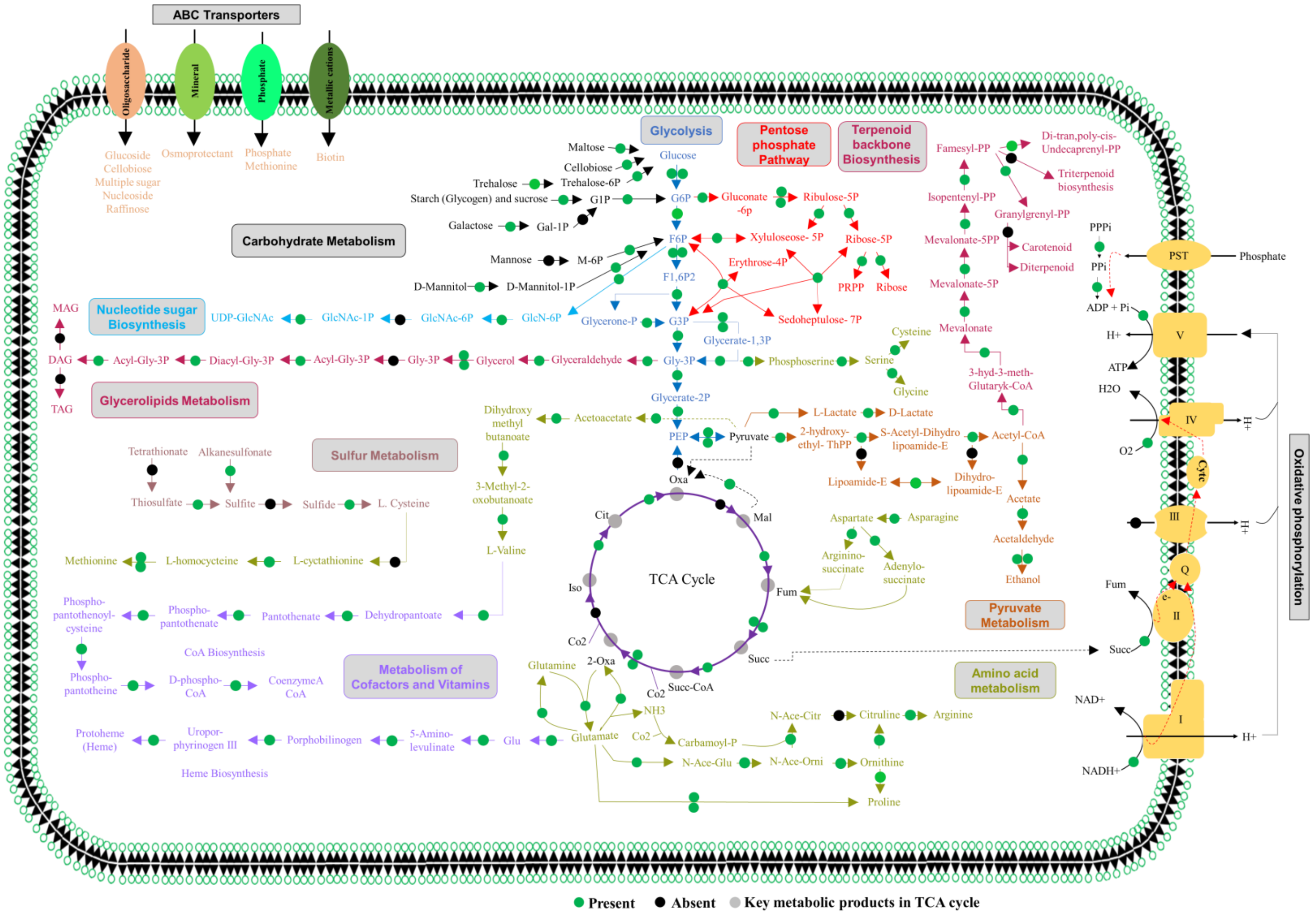

Supplementary Fig. S4. Circular visualization of the JHP9 genome , showing important functional attributes carried by it.

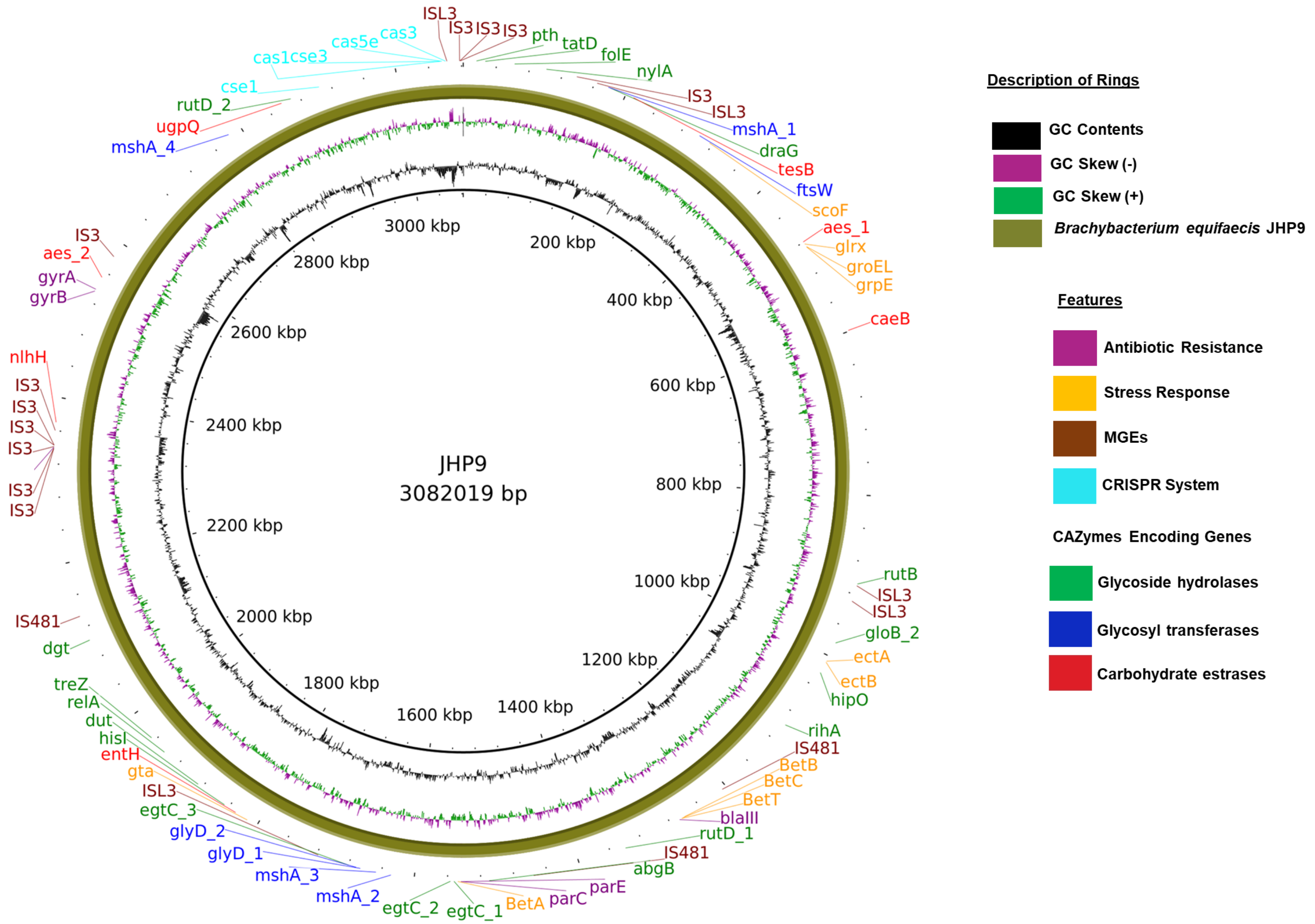

Supplementary Fig. S5. Gene clusters encoding the respiratory oxidases of the *Brachybacterium* species. These clusters encode the *bd*-type quinol oxidase and the A-type cytochrome *c* oxidase.

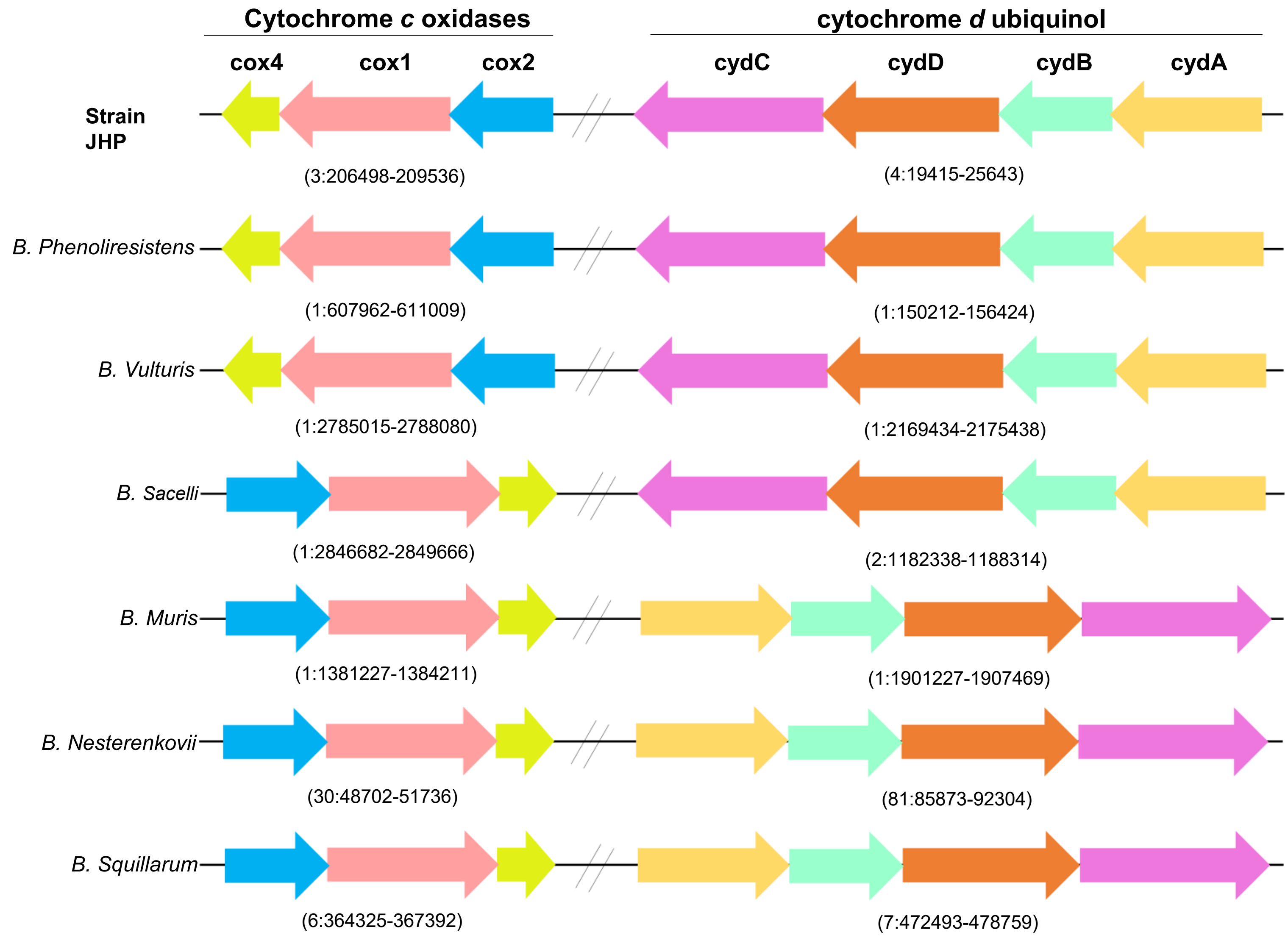

Supplementary Fig. S6. Glucose and oxygen kinetics of *Brachybacterium* strains. Michaelis-Menten plots of strain JHP9, *B. nesterenkovii*, *B. rhamnosum*, *B. huguangmaarense*, *B. horti*. Glucose and oxygen uptake rates were determined from microsensor measurements of substrate dependent O_2 consumption from either discrete slopes over many substrate concentrations (A, B, C, D, E) or a single trace measurement (F, G, H, I, J). Apparent half-saturation ($K_{m(app)}$) for glucose and oxygen were calculated by fitting the data to the Michaelis-Menten kinetic equation. The red line indicates the best fit of the data.

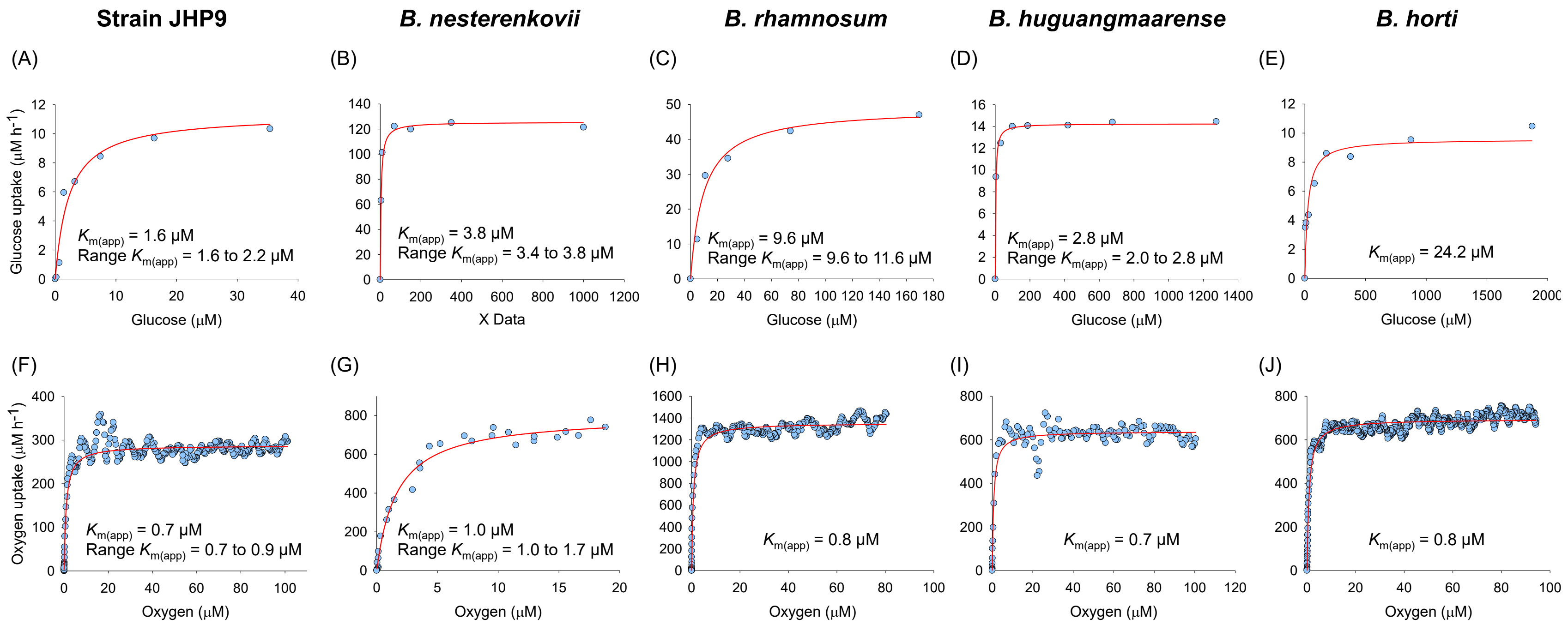

Supplement: Text S1 — Supplemental methods, tables, and figures. [file spectrum.05048-22-s0003.pdf]
